# Sodium Alginate-Based Multifunctional Hydrogel Systems for Aqueous Flexible Energy Storage: Ion Transport Regulation, Interfacial Optimization and Device Stability Enhancement

**DOI:** 10.3390/ma19214605

**Published:** 2026-10-30

**Authors:** Junyong Chen, Qingyuan Li

**Affiliations:** 1 School of Chemistry and Chemical Engineering, Qilu Normal University, Jinan 250200, China; 2 Department of Mechanical, Materials and Aerospace Engineering, Benjamin M. Statler College of Engineering and Mineral Resources, West Virginia University, Morgantown, WV 26506, USA

**Keywords:** sodium alginate, biomass hydrogel, aqueous flexible energy storage, ion transport, multi-crosslinking

## Abstract

Aqueous flexible energy storage is regarded as a highly promising safe technical route for next-generation wearable electronic devices. The central theme of this review is sodium-alginate-based hydrogel electrolytes for aqueous flexible energy storage. Among various application scenarios for such electrolytes, zinc-based flexible devices constitute the dominant research case, while supercapacitors and derivative energy-sensing integrated systems are also included for comparative analysis and extended discussion. Conventional gel electrolytes are plagued by intractable bottlenecks, including the inherent conflict between mechanical and electrochemical performances, rampant zinc dendrite growth, detrimental interfacial side reactions, narrow water-decomposition voltage window and inadequate adaptability under broad temperature conditions. Sodium alginate (SA), a cost-effective biomass polysaccharide extracted from marine brown algae, possesses distinctive structural superiority originating from the G-block “egg-box” metal-ion chelation effect for constructing high-performance hydrogel electrolytes. This review systematically summarizes three dominant modification strategies for SA-based hydrogel electrolytes and elaborates their performance enhancements together with unavoidable trade-offs. Multi-scale synergistic mechanisms are interpreted from ion-transport modulation, electrode–gel interfacial compatibility, homogeneous zinc deposition and long-term cycling stability. This work further compares SA with cellulose and chitosan, and demonstrates their applications in supercapacitors, zinc-ion hybrid energy storage, fibrous micro-devices and energy-sensing integrated systems. Critical challenges in molecular rational design and prospective large-scale manufacturing are emphasized. An integrated development paradigm of “precise molecular programming–closed-loop process control–device performance mapping” is proposed, which may offer guidance for future fundamental exploration, whereas practical engineering deployment of degradable flexible energy-storage hydrogels still requires substantial further validation.

## 1. Introduction

### 1.1. Practical Application Demands and Five Core Bottlenecks of Aqueous Flexible Energy Storage

The rapid iteration of emerging equipment, such as wearable health-monitoring terminals, flexible smart fabrics, electronic skins and implantable medical devices, puts forward practical requirements for matching energy-storage units: thin-light profile, deformability, non-toxic safety and strong environmental adaptability [1,2,3]. Traditional lithium-ion batteries suffer from intrinsic drawbacks including flammable organic electrolytes and rigid packaging and cannot withstand large-angle bending under the service conditions of flexible electronics [4].

Multiple technical routes have been proposed to realize flexible device functions. One prevailing strategy relies on mechanically bendable solid-state thin-film electrodes. Representative PES-derived laser-induced graphene (LIG) self-supporting films deliver competitive lithium-ion battery performance [5,6], while further LIG-based composite anodes and comprehensive reviews have expanded this research scope [7,8]. Even though these solid-film electrodes possess good mechanical bendability, they are primarily optimized for lithium-ion-battery working conditions. Lacking intrinsic water-retention capability and deformability-adaptive electrode–electrolyte interfacial features, such non-hydrogel configurations cannot be directly adapted to aqueous flexible zinc-based wearable energy-storage scenarios.

Under such circumstances, biomass-derived hydrogel electrolytes have aroused extensive research interest [9]. As core components of aqueous flexible energy storage, hydrogel electrolytes combine the leak-free merits of solid-state electrolytes and the high ion-conducting properties of liquid electrolytes, and can deform along with devices upon bending, stretching and compression. Hence, they have attracted extensive academic attention in recent years [10]. Nevertheless, pure-polymer hydrogel electrolytes still face intractable contradictions between electrochemical and mechanical properties, which greatly hinder the commercialization of aqueous flexible energy-storage devices. These challenges are mainly reflected in five core bottlenecks [11].

The theoretical limit of water-decomposition voltage restricts the improvement of device energy density [12]. The theoretical decomposition voltage of water molecules is merely 1.23 V, which constitutes a thermodynamic boundary for aqueous systems. Conventional unmodified hydrogels contain abundant free water molecules, which trigger hydrogen-evolution and oxygen-evolution side reactions at relatively low voltages. Accordingly, most hydrogel-based flexible energy-storage devices deliver an actual operating window below 1.5 V. Confined by such narrow voltage ranges, the output energy density of devices can hardly exceed 20 Wh·kg^−1^, a critical threshold for practical applications, creating a notable gap compared with commercial flexible lithium-based batteries. Though several studies broaden the voltage window via salt-concentration regulation or solvent replacement, such approaches usually sacrifice mechanical performance or ion-conducting capacity, making synchronous improvement of comprehensive performance difficult to achieve.

The adaptability to wide-temperature environments remains severely insufficient [13]. Abundant free-water molecules inside hydrogels crystallize below 0 °C. Formed ice crystals pierce polymer networks and cause hydrogel cracking, while ion-transport channels are blocked, leading to a sharp drop in ionic conductivity. At high temperatures or in open environments, hydrogels continuously lose water and shrink, their polymer skeletons collapse, and both mechanical and electrochemical properties deteriorate simultaneously. Anti-freezing additives such as alcohols and polyols, introduced to improve low-temperature performance, exhibit low oxidation potentials and further narrow the electrochemical stability window, resulting in performance trade-offs. Most existing hydrogels can only operate stably within a narrow range around room temperature, failing to satisfy practical scenarios such as outdoor, polar or high-temperature equipment.

Disordered zinc-dendrite growth on zinc-metal anodes serves as the primary cause of device short-circuit failure [14,15]. Zinc stands out as one of the most competitive anode materials for aqueous flexible energy storage owing to its high theoretical capacity, appropriate redox potential, abundant reserves and environmental benignity. During charge–discharge cycles, however, the uneven electric-field distribution at the electrode–electrolyte interface coupled with concentration polarization brings about local overcurrent density and sparse zinc-nucleation sites. Tiny zinc nuclei form preferentially and further develop tip effects [14]. Zinc ions keep accumulating at high-electric-field tips and grow laterally via two-dimensional surface diffusion, eventually forming rigid zinc dendrites [15]. Sharp dendrites expand continuously and directly pierce hydrogel–electrolyte separators, triggering micro-short-circuits or even complete short-circuits inside batteries, which constitutes the core obstacle limiting the long-cycle lifespan of zinc-based aqueous devices. Even without piercing, detached dendrites generate substantial “dead zinc” and continuously consume active anode materials.

Persistent interfacial side reactions deplete active zinc anodes and degrade Coulombic efficiency and cycle life [16]. Highly active free-water molecules inside hydrogels undergo continuous side reactions with zinc-metal anodes, inducing hydrogen-evolution reactions and zinc corrosion-oxidation and generating hydrogen gas, basic zinc salts and other insulating by-products. On one hand, accumulated hydrogen inside flexible packages causes bulging and potential safety hazards. On the other hand, insulating basic-zinc-salt by-products cover zinc-anode surfaces, raise interfacial charge-transfer resistance and hinder normal zinc-ion deposition-stripping. Side reactions constantly consume limited zinc anodes, reduce device Coulombic efficiency and induce irreversible capacity fading. Such phenomena become more prominent under long-term cycling and high-current charge–discharge conditions.

There exists an inherent mutually exclusive contradiction between mechanical properties and electrochemical conduction performance [17]. The mechanical strength of hydrogels largely depends on polymer cross-linking density. Higher cross-linking density markedly enhances tensile, compression-resistance and puncture-resistance capacity, yet excessive cross-linking blocks internal pores, narrows ion-transport channels and directly reduces ionic conductivity. Conversely, increasing porosity for better ion conduction yields insufficient polymer-network cross-linking, which leads to severe hydrogel swelling, sharp deterioration of mechanical strength and easy rupture under deformation. Balancing mechanical toughness and ion-transport capacity remains a key difficulty in hydrogel-electrolyte development.

Coupled with one another, the above-mentioned five bottlenecks jointly cause rapid capacity decay and poor service reliability, hindering the practical deployment of flexible aqueous energy-storage devices. Among diverse polymer substrates, multifunctional biomass-based sodium-alginate hydrogels, with unique molecular-chelating properties, tunable cross-linking networks, low raw-material costs and biodegradability, emerge as highly competitive candidates to simultaneously tackle these multiple bottlenecks [18].

### 1.2. Comparative Analysis of Mainstream Biomass-Hydrogel Matrices

Biomass polymers feature natural origins, biodegradability and favorable environmental compatibility, which conform to the development tendency of green energy-storage materials. Sodium alginate, cellulose and chitosan represent three widely applied hydrogel matrices for flexible zinc-ion batteries. As polysaccharide polymers, hydrogels prepared from all three materials commonly suffer from inherent drawbacks including non-uniform pore-size distribution, hydrophilic–hydrophobic micro-phase separation and high fractions of internal free water. These defects compress electrochemical windows and disturb zinc-ion-migration kinetics. Nevertheless, essential differences lie in functional-group species, cross-linking behaviors and electrochemical compatibility among them. A quantitative comparison of comprehensive properties is shown in Table 1.

Extracted from marine brown algae, SA carries abundant carboxyl groups (–COO^−^) along molecular chains. Such carboxyl groups possess strong electrostatic-chelating capacity and enable precise regulation of hydrogel micro-pore structures. Electrostatic interactions between ethyl viologen and SA carboxyl groups can construct continuous charge-transport pathways and effectively lower electrolyte–electrode interfacial impedance. Cumulative studies verify the outstanding comprehensive performance of SA composite hydrogels: chitosan/sodium-alginate (CTS/SA) composite hydrogels mitigate polyelectrolyte effects and achieve 0.1 MPa mechanical strength together with ionic conductivity of 2.96 × 10^−1^ S·cm^−1^. PVA/SA/MXene-NaCl double-network hydrogels reach ionic conductivity of 40.8 mS/cm and retain 88.98% of capacitance after 5000 charge–discharge cycles [28]. SA carboxyl groups can also form strong interactions with water molecules to confine internal free-water molecules, broadening the electrochemical window to 0–2.6 V. SAM–Zn systems deliver a specific capacity of 219.4 mA h g^−1^ and an energy density of 210 Wh·kg^−1^ [18].

Cellulose-based hydrogels are derived from agricultural and forestry biomass resources such as crop straws, yet natural lignin skeletons bring inherent limitations. Hydrogels prepared via conventional processes generally exhibit weak mechanical performance, limited specific capacitance and high solvent consumption during fabrication. Delignification modification removes partial lignin and hemicellulose, exposes more hydroxyl groups, enlarges internal pores and raises ionic conductivity to 38.91 mS·cm^−1^. However, a prolonged 4-h treatment only slightly elevates conductivity to 39.21 mS·cm^−1^, demonstrating that the DW-3 lignin-retaining scaffold achieves the optimal performance balance [29]. Even after delignification, cellulose hydrogels cannot eliminate hydrophilic–hydrophobic micro-phase separation. Ion-transport channels tend to fracture and restrict zinc-ion-migration rates and zinc-ion transference numbers.

From the perspective of molecular-mechanism comparison, SA builds networks via strong ionic coordination of carboxyl groups. Cellulose merely relies on hydrogen bonds among hydroxyl groups. Such hydrogen bonds are readily disrupted in aqueous environments and result in loose network structures. In terms of mechanical properties, SAM–Zn sodium-alginate hydrogels sustain 2400% tensile strain, 0.18 MPa tensile stress and 50% compressive strain (0.08 MPa) [18], which is far superior to the intrinsic brittleness of cellulose hydrogels. For electrochemical regulation, SA can realize an “ion-water-locking” effect with ionic-liquid additives to improve water retention. By contrast, cellulose reduces swelling mainly through conductive-filler doping, yet hydrophobic conductive fillers may destroy water-transport pathways; hence, cross-linking and doping ratios must be carefully balanced [30]. In terms of cost and degradability, sodium alginate boasts wide raw-material sources and moderate degradation rates. Delignification of cellulose requires complicated procedures and high solvent consumption, which increases overall manufacturing costs. Amino groups in chitosan protonate readily in acidic electrolytes and trigger local charge accumulation that distorts interfacial electric fields. Accordingly, chitosan mostly serves as an auxiliary blending component and cannot be independently adopted as a high-performance gel electrolyte for zinc-ion batteries.

Aqueous flexible energy-storage systems mainly include zinc-ion, sodium-ion and lithium-ion configurations, each of which possesses distinct advantages and application bottlenecks in wearable scenarios. To clarify the performance disparities among representative state-of-the-art works published from 2024 to 2026, Table 2 conducts cross-system comparisons covering lithium-ion, sodium-ion and zinc-ion aqueous energy-storage systems. Taking zinc-ion batteries as a typical example, the differences between hydrogel electrolyte and liquid-type ionic-liquid electrolyte are also explicitly compared from the dimensions of material composition, cycle life and energy-related metrics.

As illustrated in Table 2, selected representative 2024–2026 studies cover hydrogel/gel and ionic-liquid electrolyte systems for zinc-ion, sodium-ion and lithium-ion energy-storage. Hydrogel-electrolyte systems can eliminate liquid-leakage risks and realize mechanical compatibility for wearable devices, yet still face practical bottlenecks such as dehydration, swelling and the trade-off between mechanical performance and ion-transport behaviour. Notably, multifunctional hydrogel/gel/ionic-liquid electrolytes (e.g., PPG-Li [G], COF-reinforced AFCGE [H], ZIF-8@PAM/SA composite hydrogel [3], anhydrous HZLE ionic-liquid electrolyte [I]) can integrate flame-retardant, interfacial-regulation, sensing or high-temperature-resistant functions toward high-safety devices.

### 1.3. Unique Structural Advantages of Sodium Alginate for Constructing Functional Hydrogels

**Figure 1:**
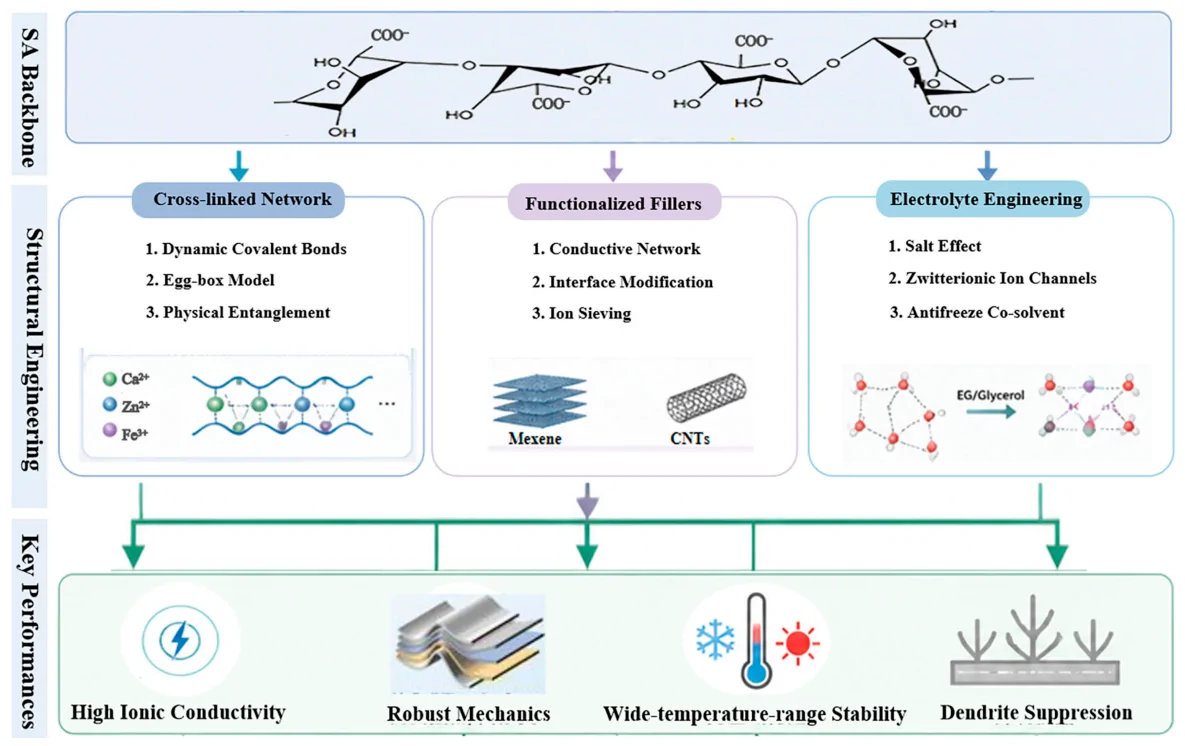
Schematic illustration of modification strategies and micro-mechanisms for SA hydrogel electrolytes. Based on the SA backbone, three modification approaches (cross-linked network construction, functionalized filler incorporation, electrolyte engineering) achieve high ionic conductivity, robust mechanics, wide-temperature-range stability and dendrite-suppression via multiple micro-effects.

Sodium alginate is a linear anionic polysaccharide polymer composed of alternating α-L-guluronic-acid (G-block) and β-D-mannuronic-acid (M-block) units. Abundant carboxyl and hydroxyl functional groups distributed along molecular chains endow it with excellent metal-ion-chelating capacity and reversible supramolecular-cross-linking properties. Specifically, G-blocks can specifically chelate multivalent metal cations such as Zn^2+^ and Sn^4+^ and self-assemble with metal ions to form the well-known three-dimensional “egg-box” cross-linking network. This molecular characteristic constitutes the core advantage unavailable to other biomass polysaccharides such as cellulose and chitosan. Figure 1 illustrates the SA molecular G/M-block structure, “egg-box” coordination effect, as well as multi-scale ion-regulation and dendrite-inhibition micro-mechanisms. Benefiting from such unique structures, sodium alginate shows prominent application potential in ion regulation, zinc-dendrite suppression and multi-component composite modification.

In terms of ion regulation, the intrinsic carboxyl groups on the sodium-alginate molecular chain exhibit electron-donating effects. In the SA-based composite system [19], Zn^2+^ capture and homogenized interfacial electric field are observed. Such phenomenon originates from SA carboxyl groups under these specific experimental conditions. Existing experiments prove that introducing carboxyl functional groups can enhance the system ionic conductivity by five times, up to 0.30 S·m^−1^. This originates from dynamic association-dissociation between carboxyl groups and free cations, which builds continuous ion-migration pathways [34]. The ζ-potential on sodium-alginate-hydrogel surfaces reaches approximately −25.4 mV. Negatively charged polymer backbones realize cation-sieving effects to repel anion migration and effectively mitigate concentration polarization [35]. Meanwhile, carboxyl groups reconstruct Zn^2+^ solvation-sheath structures and weaken Zn^2+^-H_2_O coordination-bond strength to lower desolvation barriers. Molecular-dynamics MSD simulations reveal distinctly improved Zn^2+^ diffusion coefficients with SA polymer chains, enabling efficient and directional ion transport.

For zinc-dendrite suppression, SA carboxyl-chelating effects stabilize electrode-electrolyte interfaces and restrain dendrite growth through multiple synergistic mechanisms. First, SA polymer chains preferentially adsorb onto zinc-anode surfaces to form functional interfacial protective layers, capture highly active free-water molecules at interfaces, reduce interfacial water activity and mitigate side reactions from the source [36]. Second, dynamic ionic coordination between carboxyl groups and Zn^2+^ is validated by blue-shifted −COO^−^ characteristic peaks in FTIR and XPS characterizations. Uniform zinc-ion-transport channels are constructed to equalize spatial zinc-ion flux, induce homogeneous zinc-metal deposition and suppress two-dimensional lateral dendrite growth. Featuring both ionic bonds and hydrogen bonds, SA-Zn dynamic networks simultaneously tune hydrogel mechanical rigidity and ion-transport efficiency, stabilize electrode-electrolyte contact interfaces and suppress water-decomposition side reactions under high voltage so as to broaden electrochemical-stability windows [37].

In respect of the composite-modification compatibility, plentiful carboxyl and hydroxyl groups on sodium-alginate molecules can interact with other polymers and nano-fillers via hydrogen bonds, ionic coordination and covalent grafting to build interpenetrating three-dimensional networks and simultaneously improve mechanical and electrochemical performances. Serving as a reinforcing bonding phase, SA forms strong hydrogen-bond interactions with polyaniline (PANI) conductive polymers to strengthen network mechanical performance while maintaining tight contact of active substances [38]. High-density carboxyl groups also physically entangle with gelatin triple-helix structures, or in-situ form protective layers on SnS_2_ electrode surfaces: Sn^4+^ coordinates with SA carboxyl groups to generate egg-box gel layers, achieving multiple functions including buffering volume expansion, inhibiting particle agglomeration and selective ion transport. The electrode charge-transfer resistance is reduced to 67.73 Ω, and Li^+^ diffusion coefficients rise to 10^−8^–10^−12^ cm^2^·s^−1^ [39]. Furthermore, sodium alginate has low raw-material costs, favorable film-forming processability and excellent biocompatibility, and is highly suitable for fabricating semi-interpenetrating-network gel electrolytes. Carboxyl groups also facilitate zinc-salt dissociation and stabilize three-dimensional polymer skeletons [40]. Leveraging synergistic effects of carboxyl coordination and hydrophilic-group water-retention, sodium-alginate hydrogels effectively alleviate diverse side reactions induced by aqueous environments and provide structure-function-integrated solutions for flexible energy-storage devices.

### 1.4. Research Gaps in the Existing Literature and Innovations of This Review

Multiple review papers concerning biomass and sodium-alginate hydrogels for energy storage have been published domestically and internationally, including reports in ACS, RSC and domestic core journals. However, obvious research gaps remain in current reviews. Firstly, modifications are fragmented. Preparation routes including single-network, dual/multi-interpenetrating structures, conductive-filler doping and salt-solvent regulation have not been fully integrated into a unified global analytical framework. Modification approaches are scattered across chapters without sufficient horizontal comparison or synergy-effect analysis [41]. Secondly, mechanistic interpretations are fragmented. Most reviews rely merely on SEM and electrochemical-impedance macroscopic characterizations. Few adopt DFT theoretical calculations, molecular-dynamics simulations and in-situ optical/electron-microscopy characterizations to conduct multi-scale coupled analysis. Mechanisms of ion transport, interfacial behaviors, dendrite suppression and cycle stability are discussed separately, and a unified four-dimensional collaborative mechanism covering “molecular level-mesopore-electrode interface-device performance” has not been established [15,19]. Thirdly, standardized quantitative benchmarking systems are lacking. Existing reviews mostly list experimental results in descriptive texts without comparative tables of unified indicators, making it difficult for readers to distinguish merits of different SA-modified formulas or rapidly screen material systems for specific service conditions [29]. Fourthly, most reviews are written from laboratory-material-research perspectives and pay little attention to large-scale-production realities. Industrial-grade brown-algae raw-material fluctuations, high energy consumption freeze-drying processes, high costs of high-end nano-fillers and batch-to-batch pore-structure dispersion are rarely discussed, and process-optimization schemes for pilot-scale amplification are scarce [42].

Targeting the above-mentioned shortcomings in existing publications, this review puts forward four differentiated innovations. (1) Systematically sort out preparation and modification routes of sodium-alginate hydrogels and summarize performance responses and trade-off relationships corresponding to each modification. (2) Combine multi-scale theoretical simulations, in-situ micro-characterizations and diverse electrochemical tests to construct a four-dimensional collaborative-regulation mechanism: “molecular coordination-pore-channel ion transport-interfacial electric-field-zinc-metal deposition”. (3) Establish multiple standardized performance-comparison tables to quantitatively compare ionic conductivity, cycle life, wide-temperature and mechanical indicators of various SA composite hydrogels. (4) Consider both fundamental molecular design and industrial-application perspectives, analyze gaps between laboratory research and mass production, and propose an integrated development paradigm: “precise molecular programming-closed-loop process regulation-device-performance mapping”. This work compensates for deficiencies of previous reviews which over-emphasize laboratory-scale research while lacking systematic integration, quantitative benchmarking and prospective engineering-oriented considerations.

### 1.5. Literature Retrieval and Screening Strategy

The literature for this review was retrieved from Web of Science, and Scopus, using combined keywords including sodium alginate, hydrogel electrolyte, aqueous zinc-ion battery, flexible supercapacitor, hydro-gel electrolytes. The publication time window was restricted to 2015–2026. Inclusion criteria: original experimental papers reporting sodium-alginate-based hydrogel electrolytes for aqueous flexible energy-storage devices. Exclusion criteria: pure sensor-focused manuscripts without energy-storage device validation, review articles, patents and conference abstracts without complete experimental datasets. It should be noted that the published literature inevitably contains selection bias, since positive experimental results are predominantly reported, while negative or unreleased raw laboratory data are rarely publicly available.

## 2. Modification Strategies of Sodium-Alginate-Based Hydrogels and Their Performance-Response Rules

Single-component SA hydrogels suffer from intrinsic defects such as large mechanical brittleness, limited ionic conductivity, susceptibility to freezing at low temperatures, and swelling-induced water separation during cycling, so they cannot directly satisfy the long-term service requirements of flexible zinc-ion batteries [43]. Aiming at three drawbacks: molecular network defects, insufficient electronic conduction, and unreasonable ion-solvation states, three core modification approaches have been developed: multi-crosslinked network construction, functional-filler composite, and precise electrolyte-component regulation. These strategies can be applied individually or in combination to synchronously optimize the comprehensive electrochemical and mechanical properties of hydrogels from mechanical, electronic and ionic dimensions.

### 2.1. Construction of Multi-Crosslinked Networks: Synergistic Reinforcement from Physical Entanglement to Dynamic Covalent Bonds

Pure SA single-network hydrogels are cross-linked merely via discrete metal-ion cross-linking points. Under stress, cross-linking points are prone to fracture, leading to irreversible damage to the network. Multi-crosslinking builds multi-level sacrificial-bond networks through dynamic covalent bonds, metal-ion coordination, hydrogen-bond/crystallization physical entanglement. Stress is dissipated step-by-step during deformation, simultaneously solving problems including hydrogel brittleness, swelling, and the absence of self-healing capacity. Each cross-linking system has its suitable application scenarios.

Dynamic boronate-based reversible covalent bonds represent a typical self-healing cross-linking system. Dopamine-grafted SA (SA-g-DA) cross-linked with borax forms dynamic boronate networks. The obtained hydrogel tolerates high-salt electrolytes of 3 mol·L^−1^, achieves room-temperature ionic conductivity of 119.7 mS·cm^−1^. After fracture, its mechanical-healing efficiency reaches 99.9% and ion-conduction recovery rate hits 97.3% within 15 min. Reversible fracture-recombination of dynamic bonds effectively inhibits network collapse under high-salt conditions [22]. Similarly, the SA-B-DAPPy boronate-cross-linked system relies on reversible coordination between borate groups and vicinal diols, achieving tensile strain over 800%. Conductive pathways can recover immediately after fracture, matching wearable devices subjected to frequent bending [44].

**Figure 2:**
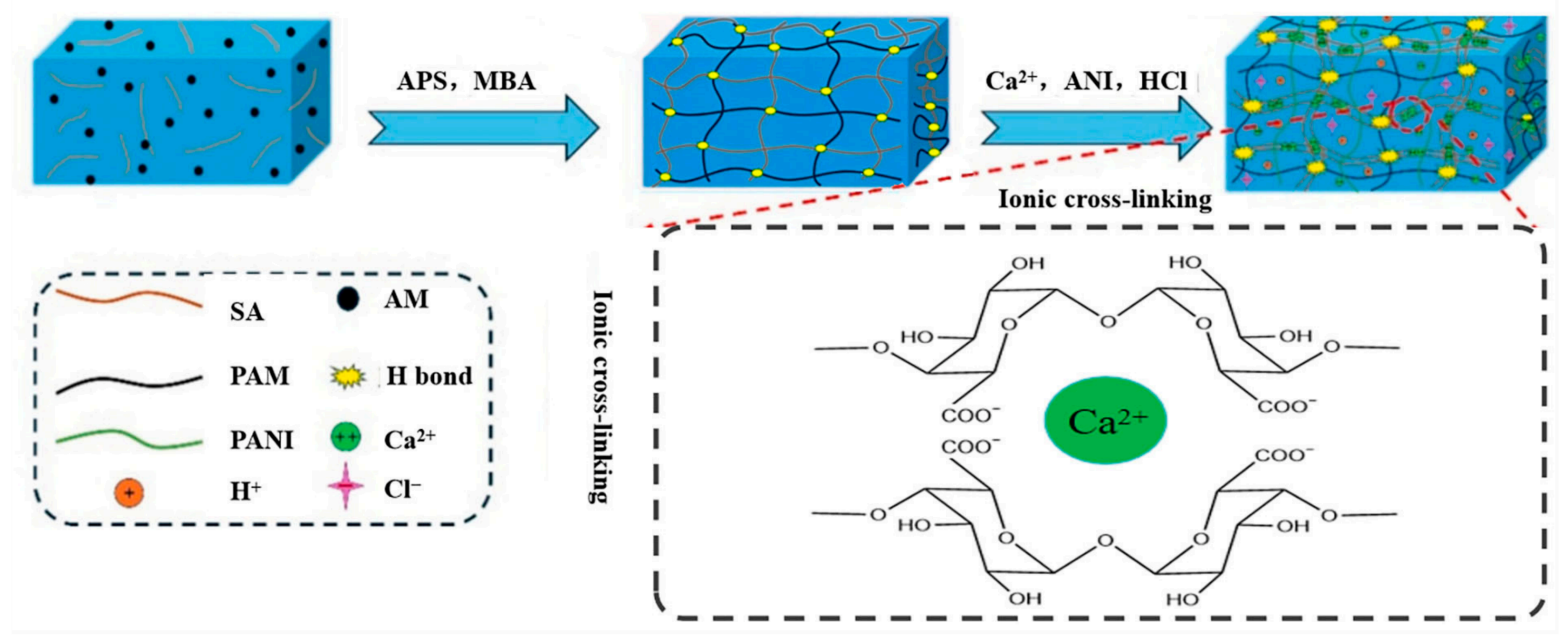
Schematic diagram of fabrication and multi-crosslinking mechanism for SA/PAM/Ca^2+^/PANI multi-network hydrogel. The synergistic hydrogen-bonding, covalent and ionic cross-linking construct multi-level sacrificial-bond networks for stress dissipation and remedying intrinsic drawbacks of pure SA single-network hydrogel.

Metal–ligand “egg-box” coordination cross-linking serves as the most mainstream modification method for energy-storage systems. Ca^2+^ specifically binds to the G-blocks of SA guluronic acid. Combined with PAM chemical main-networks and PANI conductive secondary-networks, triple-crosslinked hydrogels are constructed. The corresponding interaction mechanism is illustrated in Figure 2. The SA/PAM/Ca^2+^ ternary composite hydrogel delivers tensile strain of 1991% and tensile strength of 0.577 MPa, with room-temperature ionic conductivity of 16.51 S·m^−1^, balancing high toughness and ion conduction [45]. For zinc-based energy-storage systems, Zn^2+^-mediated cross-linking shows better adaptability. Zn^2+^ forms reversible ionic cross-linking sites with SA G-blocks, acting both as cross-linking agents and charge carriers within PAAm/SA double networks. It retains ionic conductivity of 11.08 S·m^−1^ at −20 °C and greatly improves low-temperature bending resistance [46]. Multivalent metal ions such as Fe^3+^ and Cu^2+^ can further enhance network toughness. The PXS-Fe^3+^/LiCl system relies on multiple metal-coordination sacrificial bonds and achieves fracture toughness up to 5.5 MJ·m^−3^, which substantially alleviates structural damage caused by volume expansion during charge–discharge [47].

Hydrogen-bonding and freeze–thaw-induced crystallization constitute auxiliary physical cross-linking, which are frequently compounded with ionic cross-linking [48,49]. After blending PVA with SA, freeze–thaw cycles generate crystalline physical networks. Enhanced characteristic PVA crystallization peaks can be observed in XRD patterns, with crystalline domains serving as rigid reinforcing phases. The basic PVA/SA system maintains tensile stress of 69 kPa under 114% strain [50]. In the PVA–SA–PPy composite system, hydroxyl groups of PVA, carboxyl groups of SA and amino groups of PPy form multiple intermolecular hydrogen bonds. Red-shifts of O-H and C-O characteristic peaks in FTIR confirm strong molecular interactions. The hydrogel tensile strength increases to 1.5 MPa with fracture elongation of 380% [51]. In-situ pre-dispersed NaCl homogenizes hydrogen-bond distribution and reduces crystalline agglomeration defects, so that conductive fluctuation is suppressed during repeated stretching-bending cycles [52]. In Zn^2+^-cross-linked CMC/SA composite systems, metal-coordination bonds coexist with molecular hydrogen bonds. Zn-O coordination characteristic peaks can be detected via FTIR and XPS. Cross-linked networks gain superior anti-swelling performance, and homogenized interfacial electric fields suppress the initiation of zinc dendrites [53].

Dynamic covalent bonds, metal coordination, and hydrogen-bond-crystallization cross-linking each have clear application boundaries [54,55]. Boronate-based dynamic-covalent cross-linking endows hydrogels with excellent self-healing performance, yet it is highly sensitive to pH and salt concentration. Borax itself is toxic, and the reversibility of dynamic bonds decays in high-salt electrolytes. At present, most self-healing data are only verified on small-size laboratory samples; mechanical-conductive synergistic-healing data for large-area flexible devices remain scarce. The metal-ion “egg-box” coordination is the most efficient modification for SA systems, but networks cross-linked solely by metal ions exhibit limited anti-swelling capacity under high-salt conditions. Hydrogen-bond-crystallization cross-linking features simple preparation procedures, while batch controllability is restricted by freeze–thaw cycles and temperature parameters. In general, a single cross-linking mode cannot simultaneously realize high toughness, high-salt resistance, self-healing and wide-temperature adaptability. Compound multi-crosslinking represents the mainstream development direction.

### 2.2. Introduction of Functional Fillers: Integrated Conductive Networks, Ion Sieving and Interface Modification

Pure SA is an insulating polymer and cannot conduct electrons; ion migration alone accounts for energy storage. Incorporating nano-functional fillers can simultaneously build continuous electronic pathways, construct hierarchical ion pores, and in-situ modify the SEI interface of zinc anodes. Fillers are categorized into three groups: conductive polymers, carbon-based nanomaterials, and MOF/metallic inorganic fillers.

**Figure 3:**
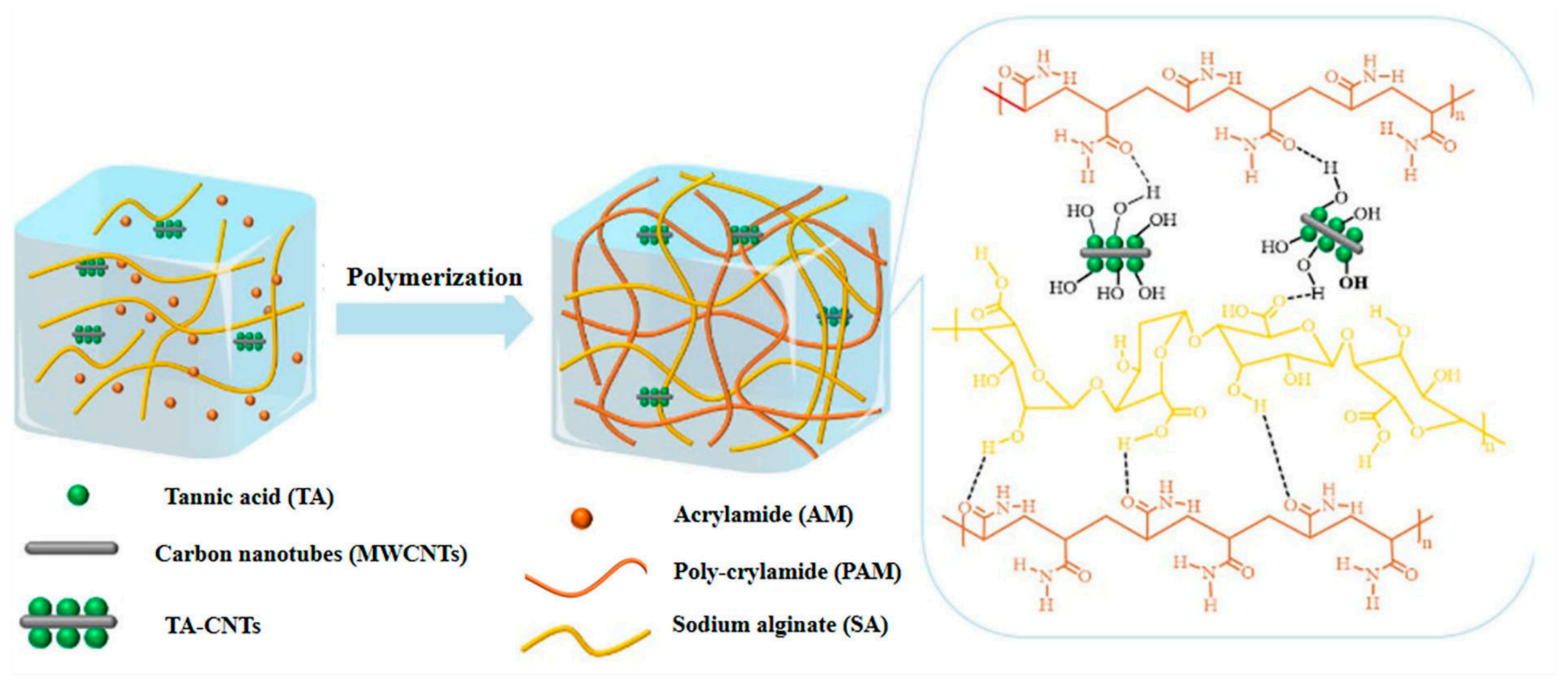
Schematic diagram illustrating the preparation of TA-CNTs and PAM/SA/TA-CNTs composite hydrogel.

Conductive polymers (PPy, PANI, PEDOT:PSS) are in-situ polymerized within the SA three-dimensional skeleton to form interpenetrating conductive fiber networks [56]. FeCl_3_-triggered in-situ polymerization produces PPy/SA nanofiber hydrogels. Continuous conductive nanofibers drastically reduce charge-transfer resistance and improve double-layer capacitance [57]. After compounding PANI with MXene and dispersing the mixture into SA matrices, sheet-shaped fillers prevent stacking of conductive polymers and expose abundant electrochemical active sites, matching high-rate energy-storage scenarios [58]. Tannic-acid-modified carbon nanotubes (TA-CNTs) disperse uniformly within PAM/SA double-network matrices. One-dimensional carbon nanotubes build long-range continuous conductive pathways and endow hydrogels with outstanding self-adhesiveness, so electrode–gel delamination hardly occurs under bending deformation [59]. The modification process of TA-CNTs and the fabrication procedure of PAM/SA/TA-CNTs composite hydrogel are illustrated in Figure 3. Tannic acid improves the compatibility of carbon nanotubes via hydrogen-bonding interactions, prevents carbon-tube agglomeration, and achieves their homogeneous distribution within the polymer network.

Carbon-based nanofillers (MXene, GO, carbon nanotubes) possess both high conductivity and negative-charge sieving effects. MXene sheets avoid stacking and form two-dimensional continuous conductive channels within SA matrices. Meanwhile, negative surface charges of MXene sheets cooperate with SA carboxyl groups to repel anions and weaken concentration polarization [60]. Negatively-charged GO sheets synergize with SA to homogenize Zn^2+^ distribution, suppress swelling-detachment of conductive polymers during cycling, and maintain long-term stable electron conduction [61,62].

MOF and metallic nanofillers focus on ion sieving and interface passivation. Doping ZIF-8 porous cage-like particles into PAM/SA hydrogels creates hierarchical ion-transport channels from internal nanopores. Ionic conductivity increases to 0.063 S·m^−1^ at a doping content of merely 1 wt% [3]. Polydopamine (PDA)-modified fillers in-situ construct stable SEI passivation layers on zinc-anode surfaces, isolating free water from metallic zinc. Interfacial resistance drops to 15 Ω, inhibiting corrosion and hydrogen-evolution side reactions over long-term cycling [63]. Even-dispersed metallic copper nanoparticles assist homogeneous zinc nucleation and further mitigate dendrite growth [64].

Although conductive polymers, carbon-based nanofillers, and MOF/metallic fillers bring multiple benefits including electronic pathways, ion sieving and SEI-interface modification, filler introduction also leads to obvious performance trade-offs [65]. Conductive polymers tend to swell and oxidize during long cycles. Two-dimensional carbon materials such as MXene exhibit excellent conductivity but suffer high cost, easy oxidation and nanosheet agglomeration. Excessive filler loading directly destroys the continuity of polymer-gel matrices and causes sharp deterioration of mechanical properties. MOF particles can build hierarchical pores, yet particle-sedimentation and poor-dispersion restrict the fabrication of thick hydrogels. Current filler-composite researches mainly focus on filler-type screening, while quantitative studies on filler-SA matrix interfacial interactions and critical optimal-doping thresholds remain insufficient.

It should be noted that conductive polymers and carbon-based nanofillers are primarily adopted to construct local interfacial conductive networks and reduce interfacial charge-transfer resistance, instead of achieving bulk electronic conduction across the entire hydrogel electrolyte. Excess filler loading may form long-range through-thickness electron pathways throughout the electrolyte, bringing potential hazards including aggravated self-discharge, electronic leakage and internal short-circuiting for zinc-based cells.

From a comprehensive perspective of conductivity, mechanical property, raw-material cost and long-term cycling stability, typical functional fillers for SA-based hydrogels exhibit obvious discrepancies. Conductive polymers (PPy, PANI, PEDOT:PSS) possess moderate conductivity and good interfacial compatibility with polymer matrix, yet they suffer from gradual oxidation and swelling upon long cycling. Carbon nanotubes deliver high conductivity and can build long-range conductive pathways, while high-quality CNTs increase material cost. MXene shows outstanding conductivity and ion-sieving capability, but it is prone to oxidation and nanosheet restacking. GO enables homogenized ion distribution and reinforces mechanical strength, though its intrinsic conductivity is relatively low. MOF fillers provide abundant ion-transport pores, yet particle sedimentation and poor dispersion limit thick-gel fabrication. Metallic nanofillers improve electrical conductivity and guide uniform metal deposition, but they may trigger partial short-circuit risk at excessive loading. Therefore, filler selection needs balanced consideration of multi-dimensional indicators rather than pursuing single-parameter optimization.

### 2.3. Electrolyte-Component Engineering: Precise Regulation of Solvation Structures via Salt Concentration, Anion Species and Co-Solvents

Abundant free water inside SA networks accounts for water decomposition, zinc corrosion and low-temperature icing. By adjusting salt loading, anion types and low-freezing-point organic-solvent ratios, the Zn^2+^ hydration sheath can be reconstructed and the proportion of free water can be reduced. Consequently, multiple goals including widened electrochemical windows, wide-temperature stability and uniform deposition are achieved. Three modification routes are available: high-concentration salt, zwitterion, and anti-freezing co-solvent.

High-concentration-salt systems confine free water through strong hydration interactions between salt ions and water molecules. Doping 30 wt% NH_4_Cl into CTS/SA composite gels weakens polyelectrolyte electrostatic association and expands internal ion pores. Room-temperature ionic conductivity reaches 2.96 × 10^−1^ S·cm^−1^, and it retains conductivity of 4.9 × 10^−2^ S·cm^−1^ at −20 °C [41]. High-concentration LiCl forms synergistic confinement structures with SA molecular chains, drastically lowering the activity of free-water molecules, suppressing high-voltage water-decomposition reactions and broadening the electrochemical-stability window [12].

**Figure 4:**
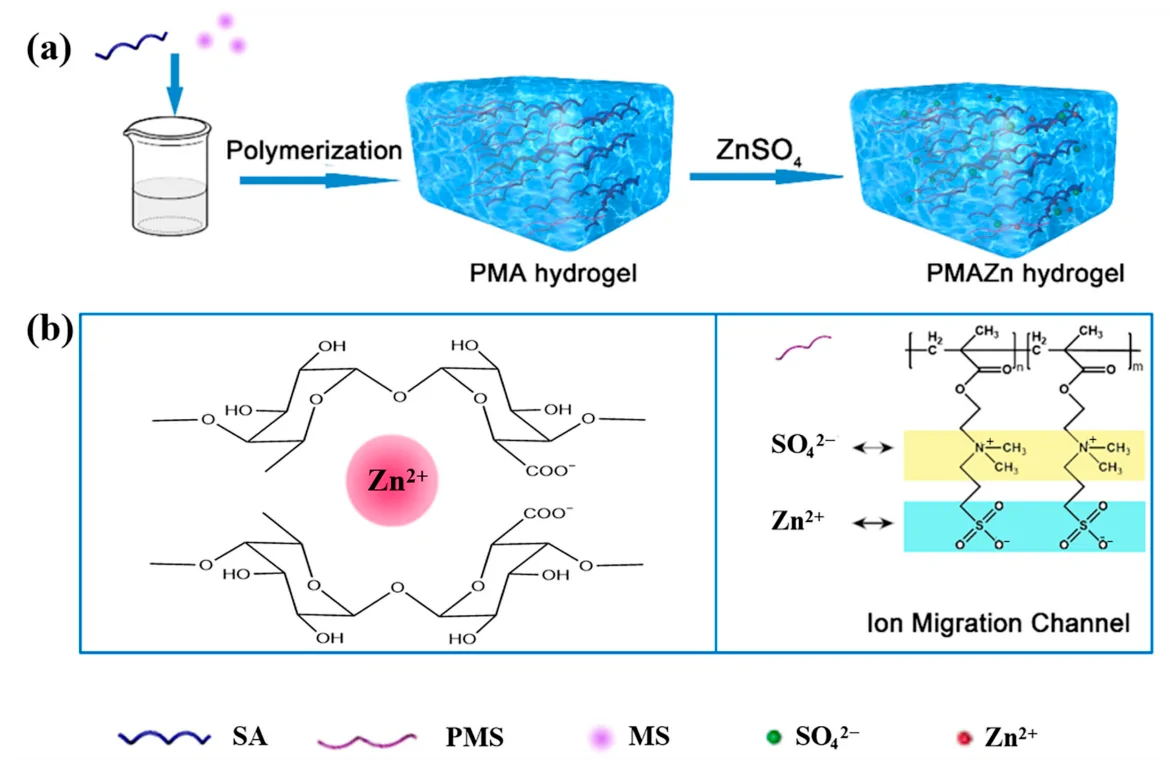
Schematic diagram of preparation and ion-regulation mechanism for PMAZn hydrogel. (**a**) Two-step synthesis of PMAZn hydrogel; (**b**) SA-Zn^2+^ ionic cross-linking and zwitterionic-polymer-based exclusive Zn^2+^ transport channels achieve selective Zn^2+^ conduction and uniform Zn deposition.

Zwitterionic anions construct exclusive Zn^2+^ transport channels via directional electrostatic interactions. When zwitterionic polymers containing SO_4_^2−^ groups are compounded with SA, sulfonate groups directionally capture Zn^2+^ and repel anions such as sulfate radicals. Tip effects induced by interfacial ion enrichment are eliminated. The device operating voltage window can be broadened to 2.5 V, and zinc is guided to deposit along the (002) crystal plane [66]. The zwitterion-mediated ion-transport regulation mechanism is illustrated in Figure 4.

Anti-freezing co-solvents such as ethylene glycol and glycerol competitively disrupt the tetrahedral hydrogen-bond networks of water molecules and lower freezing points. GG/SA/ethylene-glycol organohydrogels form mixed hydrogen-bond clusters between EG and water molecules. Ionic conductivity remains 6.19 mS·cm^−1^ at −20 °C with an electrochemical-stability window up to 2.27 V, avoiding icing failure under frigid conditions [67]. Glycerol-compounded systems simultaneously improve hydrogel toughness and water retention, reducing water evaporation under high-temperature conditions and suiting outdoor wearable devices with wide-temperature requirements [68].

Different salt species exert distinct modulation effects on SA-based hydrogel energy-storage systems. Lithium-salts improve ionic conductivity and broaden low-temperature adaptability. Ammonium-based salts adjust internal pore structure of hydrogel networks. Zwitterionic-type salts reconstruct local ion-transport microenvironment via electrostatic interaction. To further quantify these differences, Table 3 compares the influences of varied electrolyte compositions on ionic conductivity, stability, ion-transport behaviour and overall electrochemical performance.

The above-mentioned modification routes improve the comprehensive performance of SA hydrogels from cross-linking topology, electronic-conduction pathways and ion-solvation environments. Nevertheless, inherent performance trade-offs exist for each modification scheme. Multi-crosslinked networks greatly enhance hydrogel mechanical toughness and anti-swelling capacity, yet dynamic-covalent cross-linking suffers declined bond stability under high-salt and high-temperature conditions. Conductive-filler incorporation builds continuous electronic pathways and improves rate performance, but high-end nanofillers are costly; excessive loading breaks polymer-matrix continuity and degrades mechanical properties. Salt-solvent regulation reconstructs Zn^2+^ solvation-sheaths and expands operating temperature ranges, while introduced organic solvents may cause mechanical attenuation and solvent-volatilization aging.

Salt-solvent-regulation strategies effectively reconstruct Zn^2+^ solvation-sheaths to broaden voltage windows and realize anti-freezing functions, yet such modifications directly alter the micro-environment inside hydrogels. High-concentration salt intensifies polymer-chain salting-out effects and makes hydrogels brittle. Zwitterion modification optimizes zinc-deposition behavior, while some zwitterions feature high viscosity and increase processing difficulty. Anti-freezing co-solvents such as ethylene glycol and glycerol suppress ice-crystal generation, yet excessive organic solvents degrade hydrogel mechanical strength and bring potential solvent-volatilization-aging risks. Notably, complex synergistic/antagonistic interactions exist between salt-solvent components and SA molecular chains. Most studies focus on final electrochemical outputs, whereas systematic discussions on how salt and solvent change the intrinsic structure of SA “egg-box” cross-linked networks remain scarce.

Besides material-level trade-offs mentioned above, attention should also be paid to the reliability of reported electrochemical-stability-window data originating from different testing protocols. It should be emphasized that most reported electrochemical-stability-window values of hydrogel electrolytes are acquired by linear sweep voltammetry (LSV) using inert electrodes (stainless-steel, titanium or platinum), instead of realistic Zn-based full-cell configurations. Such inert-electrode test results offer idealized upper-limit reference values, which are frequently broader than the practically available voltage window in real zinc-based devices. Multiple experimental factors strongly affect the obtained stability-window results. The adoption of zinc metal anodes introduces intrinsic corrosion and hydrogen-evolution side reactions, compressing the practical usable voltage range. A higher LSV scan rate may kinetically suppress side-reaction currents and over-estimate the stable window, while a low scan rate is more representative of real-service conditions. In addition, different current-density cutoff criteria for judging water-decomposition onset will produce diverging window readings. Therefore, LSV results based on inert electrodes should be treated cautiously as reference benchmarks, and practical device-level voltage performance is recommended for reliable evaluation.

## 3. Core Regulation Mechanisms of Electrochemical Performance

Relying on molecular coordination, porous pore channels and interfacial interactions, SA-based hydrogels form a multi-scale synergistic regulation system, which addresses inherent drawbacks of aqueous energy storage from four dimensions: ion transport, electrode interface, zinc-deposition behavior and cycling durability.

### 3.1. Precise Construction and Regulation of Internal Ion-Transport Channels

**Figure 5:**
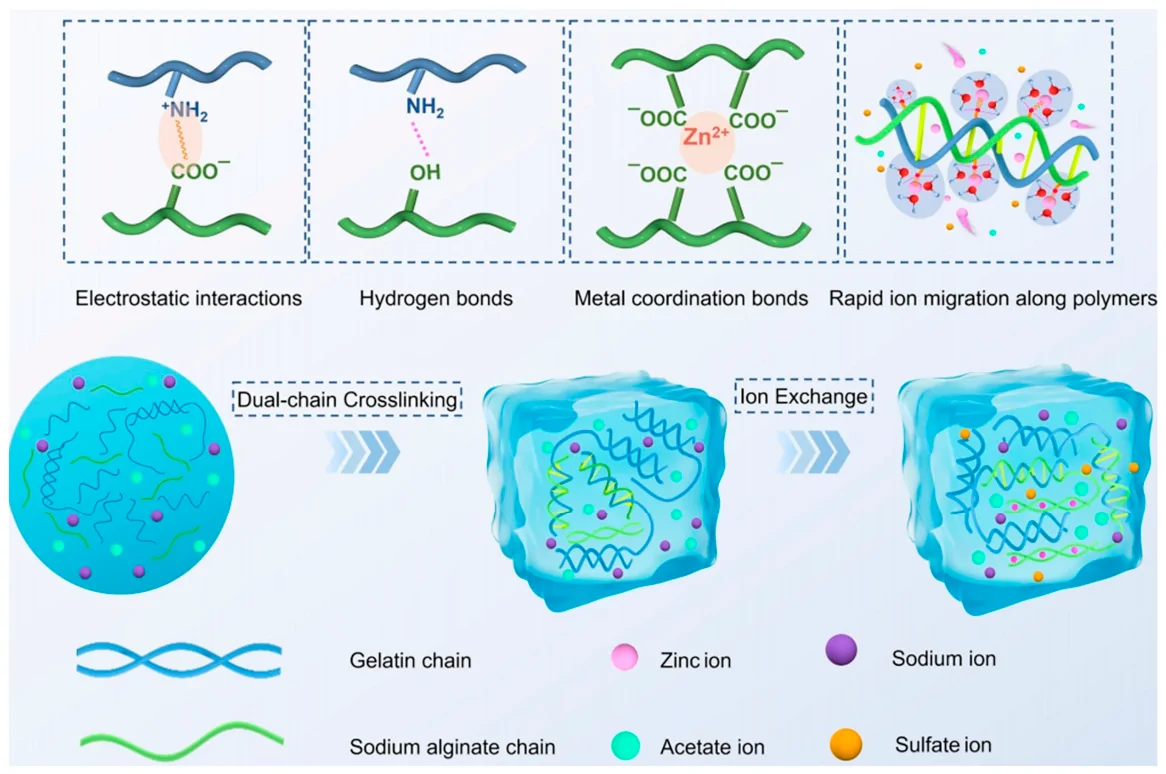
Schematic diagram of dual-chain cross-linking and ion-exchange mechanism of Gel/SA composite hydrogel. Electrostatic interaction, hydrogen bonds and metal coordination realize dual-chain cross-linking, and ion-exchange accomplishes Zn^2+^ desolvation and fast ion transport.

Ion-migration rate is jointly determined by Zn^2+^ solvation configuration and hydrogel pore-channel structure. Carboxyl groups on SA molecular chains can reconstruct zinc-ion hydration sheaths at the molecular scale and greatly reduce desolvation energy barriers. Tian et al. [19] carried out experimental characterizations and multi-scale theoretical simulations on Gel/SA-acetate double-network hydrogels. ATR-FTIR (ex-situ characterization, indirect experimental evidence) tests observed a blue-shift of the asymmetric stretching vibration peak of carboxyl groups to 1632 cm^−1^, directly confirming coordination interactions between Zn^2+^ and −COO^−^ groups. The reconstruction of Zn^2+^ solvation sheath described below is a mechanistic interpretation mainly supported by molecular-dynamics and DFT theoretical simulations, which cannot be directly visualized by experimental measurements: Molecular dynamics simulations show that SA polymer chains can intrude into the initial hexahydrated solvation sheath of Zn^2+^ and replace partial coordinated water molecules, lowering the Zn^2+^ coordination number from 6 to 3. Molecular-orbital calculation results reveal that the highest occupied molecular-orbital energy level of SA is markedly higher than that of pure water, making it easier to donate electrons to Zn^2+^ and form thermodynamically more stable complexes. DFT calculations comparing the binding energies of Zn^2+^-SA and Zn^2+^-H_2_O demonstrate stronger interaction between SA and zinc ions. The corresponding desolvation energy is only 1.36 eV, far lower than 16.33 eV in pure-electrolyte systems. Fitted by the Arrhenius equation, the ion-migration activation energy of this system reaches 25 kJ·mol^−1^ (derived from macroscopic electrochemical testing, indirect evidence for ion-transport behavior). The micro-mechanism of chain-liquid synergistically-regulated ion transport is illustrated in Figure 5.

For PAM/SA composite hydrogels, shifts of −COO^−^ characteristic peaks are also observed in FTIR spectra (ex-situ characterization, indirect evidence for Zn^2+^-carboxyl coordination), which further verify coordination behavior between Zn^2+^ and carboxyl groups [14]. The tridentate-ligand binding mode described below comes from DFT simulation prediction rather than direct experimental observation: DFT calculations prove that SA can bind zinc ions as a tridentate ligand. Meanwhile, interconnected hierarchical porous channels of 1–3 μm are formed inside the hydrogel to provide pathways for continuous ion migration. Consequently, this system achieves a high Zn^2+^ transference number of 0.86 and ionic conductivity of 5.4 S·m^−1^ (macroscopic electrochemical performance, indirect reflection of ion-transport properties).

### 3.2. Optimization of Electrode-Hydrogel Interfacial Compatibility

**Figure 6:**
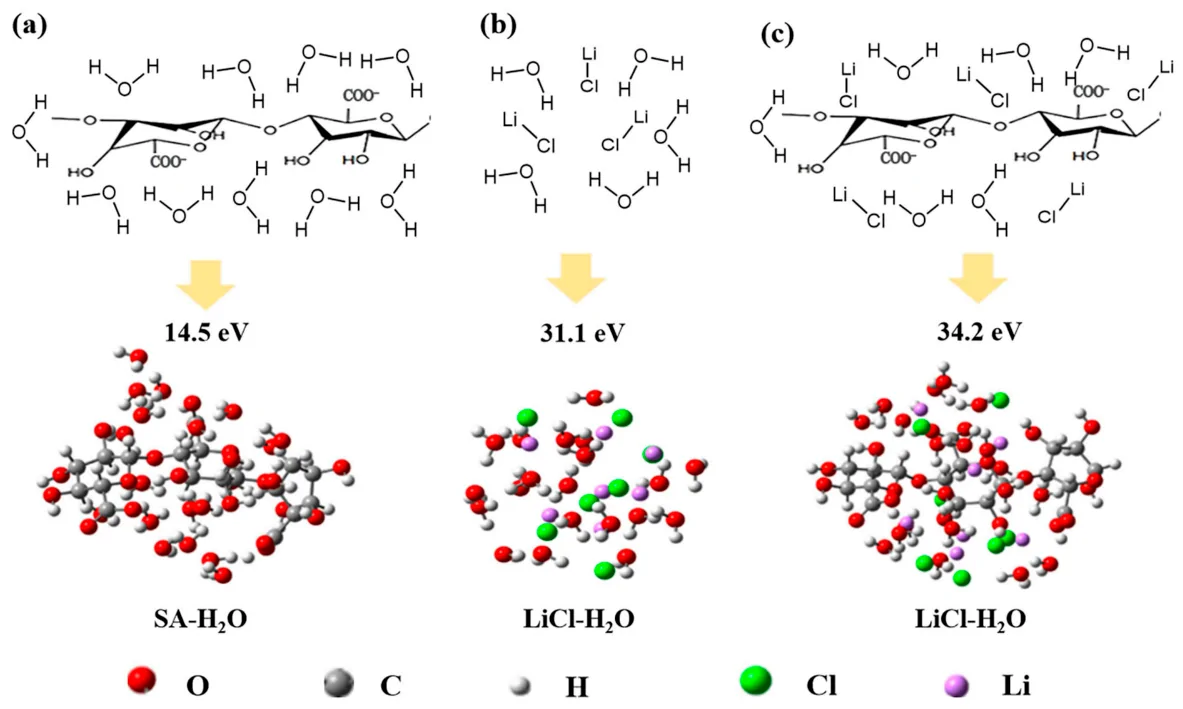
Comparison of DFT-optimized configurations and water-molecule binding energies for different systems. SA-H_2_O (**a**), LiCl-H_2_O (**b**) and SA-LiCl-H_2_O (**c**) systems are compared to demonstrate spatial-confinement effect of SAL hydrogel on water molecules.

The wettability and binding force at hydrogel-zinc-anode interfaces directly determine interfacial impedance and stability under repeated deformation. SA realizes complete zinc-surface wetting and void-free adhesion via dual effects of hydrogen bonds and metal coordination. Yu et al. [12] prepared SAL-type SA hydrogels, which induce polymer-chain aggregation to form flat surfaces, realize uniform dispersion of Cl^−^ and guarantee sufficient ion-exchange capacity of the system. Shifts of hydroxyl and carboxylate characteristic peaks are detected by FTIR. XPS tests detect elevated HO-C=O binding energy (ex-situ post-cycling characterization, indirect evidence for interfacial hydrogen-bond interactions), which directly confirms hydrogen-bond generation between SA and metal ions. The spatial water-confinement mechanism stated below is mainly supported by DFT theoretical simulation: DFT calculation results indicate that water-molecule binding energy in SA–water–metal-ion composite systems is significantly higher than that of single components. Relevant theoretical-calculation models are shown in Figure 6. Benefiting from spatial-confinement effects, free-water molecules are effectively confined. The device retains 92.3% of its initial capacity after 300 cycles of 180° repeated bending (macroscopic device cycling performance, indirect reflection of interfacial stability) [53].

SA/CMC/THL ternary composite hydrogels possess 3D interconnected porous networks. EDS elemental mapping demonstrates uniform distribution of C, O and Zn elements across the whole area. Contact-angle tests verify superior zinc-metal wettability of this hydrogel compared with liquid ZnSO_4_ electrolyte (direct experimental observation for surface wettability). Zn-O coordination-bond characteristic peaks are jointly detected by FTIR and XPS (ex-situ indirect evidence for interfacial coordination). The preferential occupation of hydrogen-evolution active sites described below originates from DFT computational prediction: DFT calculations show that SA, CMC and THL exhibit stronger adsorption energy on the Zn (002) crystal plane than water molecules, so they preferentially occupy hydrogen-evolution active sites. Calculated via steady-state polarization equations, the zinc-ion transference number of this system reaches 0.70, higher than 0.52 of pure ZnSO_4_ electrolyte. It effectively mitigates concentration polarization and optimizes uniform interfacial-ion distribution [53].

Various SA-based composite hydrogels adopt different strategies to improve electrode-hydrogel interfacial compatibility, including constructing coordination interaction, building hierarchical porous channels and regulating solvation sheath. Representative systems are summarized in Table 4, comparing their modification approaches, experimental-simulation evidence and interfacial performance.

As shown in Table 4, hydrogen-bond and metal-ion coordination are the dominant driving forces to realize favorable electrode-hydrogel contact for SA-derived systems. Hierarchical porous structures further facilitate ion migration. It should be noted that most interfacial characteristics are obtained from ex-situ characterization and theoretical simulation; real-time operando evidence at electrode–gel interfaces is still limited.

Beyond basic interfacial contact and SEI construction, dendrite growth is a typical interfacial-derived failure phenomenon closely associated with electrode-hydrogel compatibility. Significant divergences exist in dendrite nucleation, growth features and failure risks among hydrogel-based zinc-ion energy-storage systems. It is necessary to clarify their distinctions as well as the inhibition effect of sodium-alginate hydrogel matrices. Table 5 compares dendrite-related behaviours and remaining challenges for these zinc-ion configurations.

To sum up, dendrite risk originates from interfacial ion-flux inhomogeneity. Even with identical sodium-alginate hydrogel electrolytes, zinc-metal anodes still suffer dendrite risks under harsh cycling conditions, which imposes differentiated requirements for interfacial modification strategies.

### 3.3. Inhibition Mechanism of Zinc-Dendrite Growth

**Figure 7:**
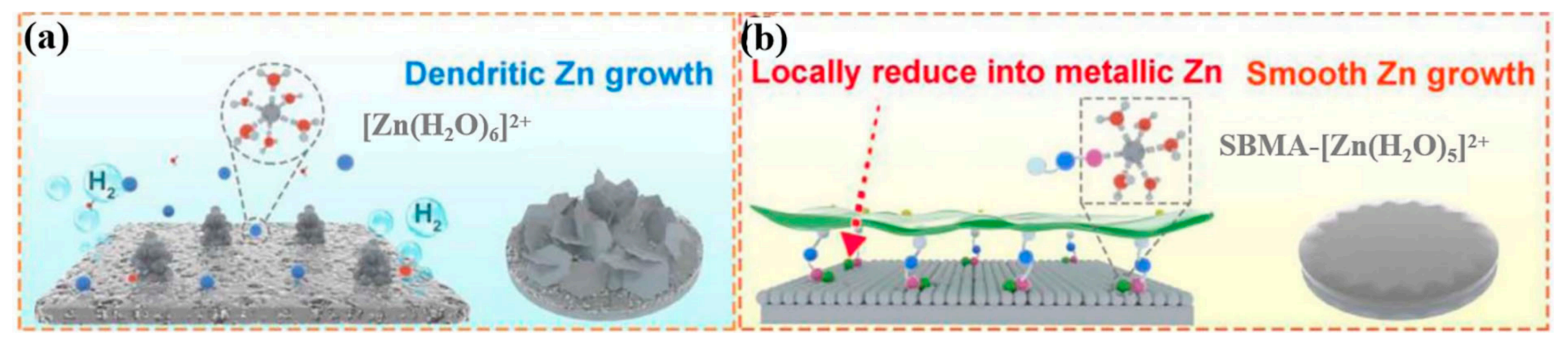
Multi-scale dendrite-suppression mechanism of SPS-Zn hydrogel. DFT adsorption-energy calculation, CA measurement, in-situ optical observation and finite-element simulation collectively reveal the inhibition of 2D lateral-diffusion (**a**) and homogenization of interfacial Zn^2+^ flux (**b**).

Zinc-dendrite growth originates from uneven electric-field distribution at zinc-anode interfaces and two-dimensional lateral-diffusion behavior of Zn^2+^. SA-based hydrogels guide uniform vertical zinc-ion deposition through three-fold synergistic effects: coordination adsorption, homogenized electric-field regulation and three-dimensional physical confinement. Li et al. [14] pointed out that abundant carboxyl groups on SA molecular chains anchor free Zn^2+^ via ionic-coordination interactions, reconstruct ion-transport environments at zinc-anode interfaces and alleviate local Zn^2+^ enrichment, laying structural foundations for dendrite suppression (mechanism inferred from ex-situ spectroscopy and cell cycling performance, without direct real-time observation). Wu et al. [15] constructed zwitterion-modified SPS-Zn hydrogels. DFT theoretical calculations show that the adsorption energy of hydrogel components on the zinc (002) crystal plane reaches −0.919 eV, remarkably higher than −0.408 eV of water molecules (simulation-derived adsorption-energy result, computational evidence). The hydrogel preferentially adsorbs onto zinc-metal surfaces and restrains unregulated two-dimensional lateral diffusion of Zn^2+^; this mechanistic interpretation is deduced from computation and electrochemical responses and cannot be directly imaged by experiments. Relevant theoretical models are presented in Figure 7. Chronopotentiometry (CA) measurements can intuitively reflect differences in ion-diffusion modes: current continuously rises up to 180 s in liquid-electrolyte systems, corresponding to long-range two-dimensional surface-diffusion behavior of Zn^2+^; by contrast, current rapidly reaches a steady state within 60 s in SPS-Zn hydrogel systems, and zinc ions switch to three-dimensional bulk nucleation modes (electro-analytical testing, indirect reflection of nucleation-diffusion mode). In-situ optical microscopy real-time monitors morphological evolution of zinc electrodes during electroplating (in-situ/operando direct visual experimental evidence): abundant dendrite bulges emerge within a short time under liquid-electrolyte conditions, while zinc-electrode surfaces stay flat and dense in SA-based composite-hydrogel systems. The homogenized interfacial electric-field mechanism below comes from finite-element simulation and serves as a proposed explanation for the observed dendrite-free morphology: Finite-element electric-field simulations further confirm that carboxyl groups on SA chains uniformly capture Zn^2+^, weaken the “tip effect” caused by local high electric fields on electrode surfaces, equalize interfacial zinc-ion flux and fundamentally curb continuous zinc-dendrite proliferation.

### 3.4. Promotion Strategy for Long-Term Cycling Stability

**Figure 8:**
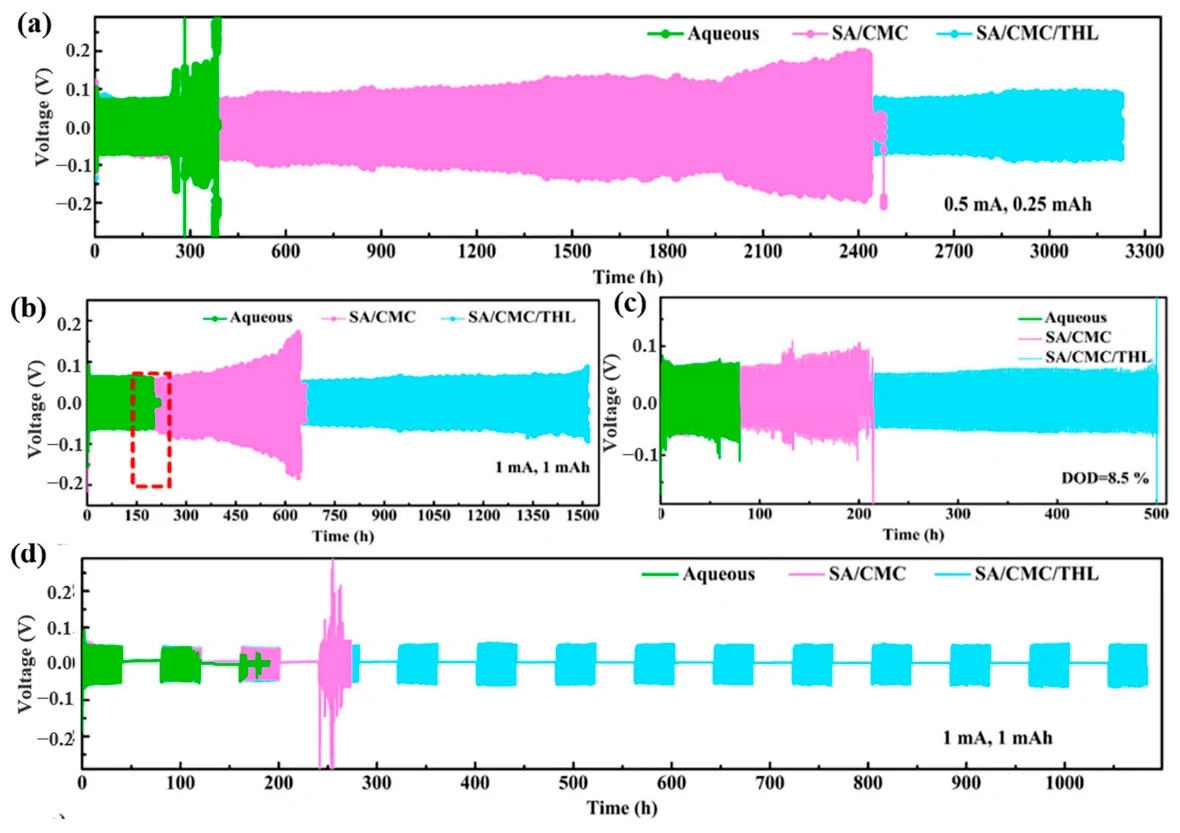
Long-term cycling comparison of Zn||Zn symmetric cells using aqueous electrolyte, SA/CMC and SA/CMC/THL gels, verifying the enhancement of cycle life from THL cross-linked network. (**a**) Cycling performance at 0.5 mA (0.25 mAh); (**b**) Cycling performance at 1 mA (1 mAh), the red dashed box marks the stage of explosive dendrite growth, severe voltage fluctuation and out-of-control polarization for aqueous electrolyte; (**c**) Cycling performance at 1 mA with a depth of discharge (DOD) of 8.5%; (**d**) Rate cycling test at 1 mA (1 mAh).

The fading of capacity during device cycling mainly stems from three failure pathways: hydrogel water-loss-induced swelling, hydrogen-evolution corrosion of zinc anodes and dendrites piercing through separators. By constructing cross-linked water-locking networks combined with zwitterionic flux regulation, bulk-structure stability of hydrogel electrolytes and electrode-hydrogel interfacial stability can be maintained for long durations. Liu et al. [49] indicated that the water loss of SA-based hydrogel electrolytes would directly destroy three-dimensional cross-linked skeletons and trigger interfacial-contact deterioration and aggravated side reactions. Appropriate cross-linking modification can strengthen water-molecule anchoring effects, suppress water volatilization and mitigate hydrogen-evolution side reactions of zinc anodes, laying foundations for long-term cycling. Zhang et al. [53] developed SA/CMC/THL composite hydrogels, which build dense cross-linked networks relying on multi-functional-group THL. TGA tests reveal markedly reduced water mass loss below 100 °C for this hydrogel. Strong intermolecular bonds effectively confine free-water molecules and disrupt ice-crystal structures, realizing electrochemical stability over a wide temperature range. The corresponding Zn||Zn symmetric cell achieves ultra-long stable cycling up to 3224 h at 0.5 mA·cm^−2^, and the average Coulombic efficiency of Zn||Cu half-cells reaches 99.42%. Long-cycling comparisons of symmetric cells adopting different electrolyte systems are shown in Figure 8.

Apart from cross-linked water-locking strategies, zwitterion modification also improves long-cycle performance from the perspective of ion transport. The zwitterionic SPS-Zn system increases uniform-nucleation sites and restricts two-dimensional lateral diffusion of Zn^2+^ via strong affinity between sulfonate groups and zinc. DFT calculations verify thermodynamically stable hydrated-ion configurations and realize uniform interfacial electrostatic-potential distribution. Symmetric cells stably cycle for 380 h at 1 mA·cm^−2^. Full cells matched with HCNFs cathodes complete 12,000 charge–discharge cycles at 3 A·g^−1^, with a single-cycle capacity decay of merely 0.0017%, achieving ultra-long service life [15].

In summary, electrochemical performances of SA-based hydrogel electrolytes result from the coupling and synergy of the above-mentioned multiple mechanisms. Single regulation mode bears obvious drawbacks, and practical systems usually require joint contributions from multiple mechanisms. These intrinsic molecular-mesoscale mechanisms are finally reflected in various service indicators of flexible energy-storage devices. The following section analyzes performance boundaries and mechanical reliability of devices from the perspective of device integration.

## 4. Integrated Application and Performance-Boundary Analysis of Flexible Energy-Storage Devices

Benefiting from high ionic conductivity, regulated uniform zinc deposition, excellent mechanical flexibility and wide-temperature adaptability, SA-based composite hydrogels are applicable to various flexible energy-storage systems, including carbon-based symmetric supercapacitors, zinc-ion hybrid supercapacitors, fibrous micro-wearable devices and integrated energy-sensing equipment. This chapter quantitatively sorts out the upper performance limits and existing drawbacks of various devices from the perspectives of comprehensive electrochemical indicators and mechanical service reliability and establishes a complete performance-evaluation system.

### 4.1. Electrochemical Performance Map of Full Devices: Energy/Power Density, Cycle Life and Environmental Adaptability

Energy density, power density, cycling stability and extreme-temperature tolerance constitute four core indicators for evaluating the practical application value of flexible energy-storage devices [69,70,71].

In terms of energy density and power density, Li et al. [72] constructed all-SA-based integrated quasi-solid-state supercapacitors. Combined with nitrogen-doped porous-carbon electrodes and homogeneous SA gel electrolytes, the device delivers an energy density of 20.2 Wh·kg^−1^ together with a power density of 112.5 W·kg^−1^. Badawi et al. [73] prepared ACODS biomass composite-gel electrolytes. With dual-network reinforcement and carbon-filler modification, the device energy density is raised to 28.4 Wh·kg^−1^. Wang et al. [74] developed ethylene-glycol-modified anti-freezing PAM-SA hydrogel electrolytes for zinc-ion hybrid supercapacitors, achieving a room-temperature energy density of 117.0 Wh·kg^−1^ with outstanding low-temperature resistance. Tian et al. [19] reported Gel/SA-acetate systems with efficient zinc-ion desolvation, reaching an energy density of 236.8 Wh·kg^−1^. Darwish et al. [75] fabricated rGO-composite symmetric supercapacitors with a peak power density of 37.49 kW·kg^−1^.

There exists an inherent trade-off between power density and energy density. Improving power output usually sacrifices energy density, and it is difficult to optimize both simultaneously [76,77,78]. Ruthes et al. [79] fabricated flexible supercapacitors with a power density of 19.9 W·kg^−1^ but only 3.0 Wh·kg^−1^ energy density. After heavy-load bending, the maximum specific-capacitance decay of the device reaches 88%, reflecting the strict requirements of flexible service conditions. SA-based gel energy-storage systems also reveal obvious limitations in performance trade-offs.

Multiple modified SA gel devices also exhibit competitive energy output performance [80,81,82]. Huang et al. [83] obtained 164.13 Wh·kg^−1^ for SPMA-ZHS devices; Liu et al. [84] reported an energy density of 53.00 Wh·kg^−1^ for NCBS/C//AC asymmetric devices. Restricted by electrode structures and intrinsic gel properties, some systems cannot meet practical requirements. Badawi et al. [85] reported Na-Alginate/PEDOT:PSS symmetric devices with a gravimetric energy density of 40.58 Wh·kg^−1^.

In terms of cycle life, numerous modified SA-based systems realize stable charge–discharge for more than ten thousand cycles [86]. The quasi-solid-state supercapacitor totally derived from sodium alginate delivers a capacitance retention of 95.9% after 10,000 cycles at a current density of 10 A·g^−1^ under room-temperature conditions [72]; the fibrous supercapacitor based on GO/NH_2_-UiO-66/SA composite hydrogel electrolyte achieves a capacitance retention of 91.7% after 11,000 charge–discharge cycles at 0.446 mA·cm^−2^ [87]; the zinc-ion hybrid supercapacitor based on freeze-tolerant PAM-SA gel electrolyte achieves a capacity retention of 65% after 40,000 charge–discharge cycles at a current density of 2 A·g^−1^ under room-temperature conditions [74]; the asymmetric supercapacitor based on SA/PVA/PAA-KOH hygroscopic hydrogel electrolyte attains a capacitance retention of 92.6% after 10,000 charge–discharge cycles under 40% relative humidity and 32 mA·cm^−2^ [88]; the Zn@CC|SAP-1.5 zinc-ion hybrid supercapacitor delivers a capacity retention of 94.6% after 10,000 charge–discharge cycles at a current density of 1 A·g^−1^ under 25 °C [46]; the zinc-ion hybrid supercapacitor based on zwitterion-modified SPS-Zn hydrogel electrolyte exhibits nearly negligible capacity decay over 12,000 charge–discharge cycles at a current density of 3.0 A·g^−1^ [15].

Environmental adaptability directly determines application scenarios including outdoor and low-temperature wearable conditions. GO/NH_2_-UiO-66/SA fibrous devices operate within −40~40 °C and retain 79.1% capacitance at −40 °C [87]; phase-change composite-gel devices exhibit resistance fluctuation of only 2.11 Ω from −20 °C to 60 °C [89]; SALs-3 devices work stably from −20 °C to 50 °C and retain 92.1–99.2% capacity after 5000 low-temperature cycles [12]; zinc-activated-carbon asymmetric devices keep 69% capacity at −30 °C [81]; Zn@CC|SAP-1.5 retains 90.7% cycling capacity at −20 °C [46]; SSC-PVA/GO systems adapt to the full temperature range of −20~60 °C [90]. In contrast, PVA/SA/MXene systems lack low-temperature performance data [91]; most micro-supercapacitors have not undergone extreme sub-zero-temperature tests [92]; all-solid-state flexible devices are only validated at −15 °C without further exploration for frigid environments [93].

Device integration with various SA-based gel electrolytes can construct multiple configurations of aqueous flexible energy-storage devices. Constrained by electrode materials, gel formulas and device-packaging modes, devices differ greatly in energy density, cycle life, temperature resistance and mechanical tolerance. Several systems achieve simultaneous high-energy output and stable long-term cycling, whereas fibrous micro-devices suffer obvious energy-density drawbacks due to limited active-material loading. Key indicators of typical devices are summarized in Table 6 for clear comparison of performance boundaries for different device configurations.

As shown in Table 6, zinc-ion hybrid supercapacitors deliver the best comprehensive performance with balanced high energy density and wide-temperature-range capability. Carbon-based symmetric supercapacitors exhibit outstanding cycling stability yet generally limited energy density. Fibrous and micro-wearable devices satisfy miniaturization and weaving requirements but suffer low energy density originating from insufficient active-material loading. Apart from electrochemical indicators, flexible wearable devices continuously undergo bending, stretching, compression and other external forces in practical service. The intrinsic toughness of gels and interfacial binding strength between electrodes and gels directly determine service reliability. The mechanical service reliability of devices is analyzed below.

**Figure 9:**
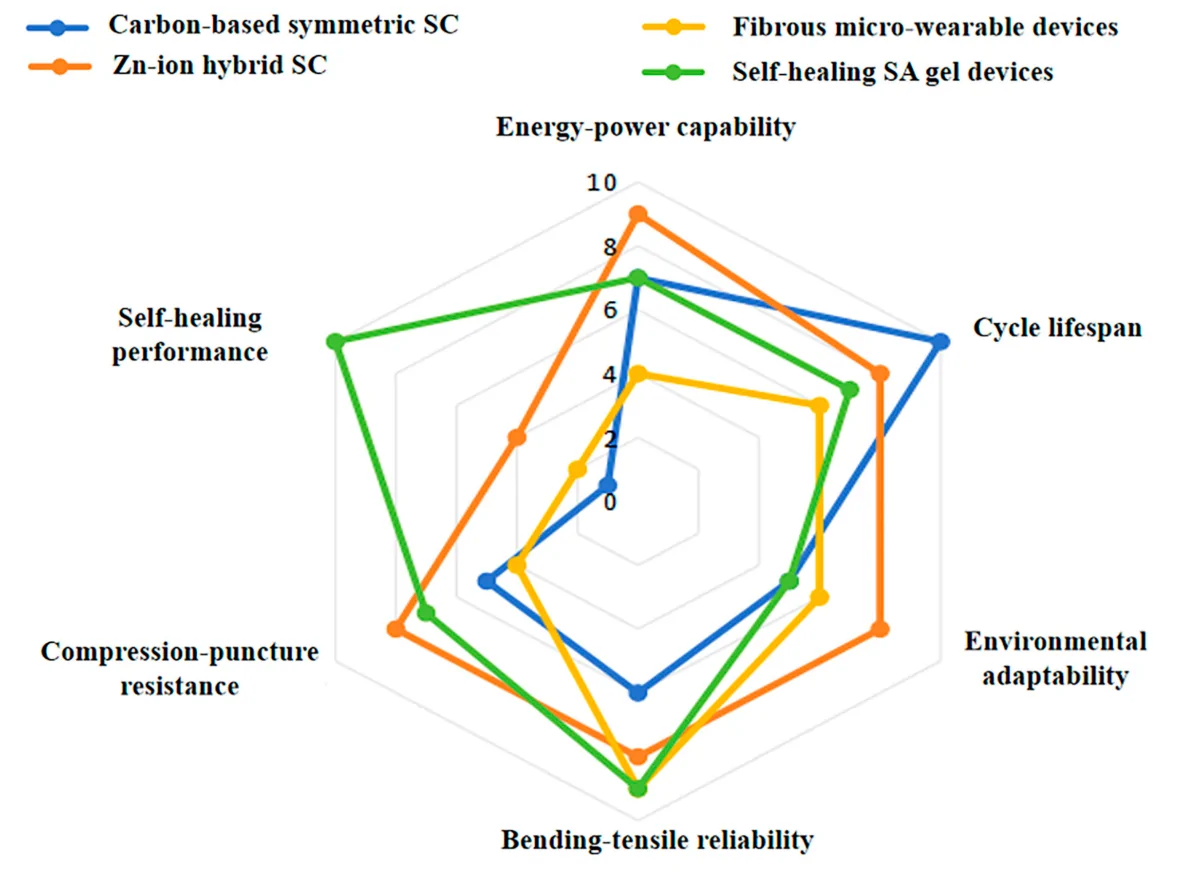
Radar-chart multi-dimensional performance comparison of typical SA-based hydrogel flexible energy-storage devices.

To intuitively compare the comprehensive performance of various SA-based hydrogel flexible energy-storage devices, a multi-dimensional radar chart is constructed (Figure 9). Six evaluation dimensions including energy-power capability, cycle lifespan, environmental adaptability, bending tensile reliability, compression-puncture resistance and self-healing performance are selected, and all indicators are normalized into a score range of 0–10. As illustrated in Figure 9, zinc-ion hybrid supercapacitors show balanced and superior overall performance with excellent energy-power output, wide environmental adaptability and good mechanical tolerance. Carbon-based symmetric supercapacitors possess the optimal cycle lifespan, but their self-healing capability is poor. Fibrous micro-wearable devices exhibit excellent bending tensile reliability, while their energy-power capability and compression-puncture resistance are relatively weak. Self-healing SA gel devices achieve outstanding self-healing performance and favorable mechanical reliability, yet their energy-power performance and environmental adaptability remain moderate.

### 4.2. Flexibility and Reliability Verification: Stability Under Bending, Stretching, Compression and Self-Healing Conditions

In practical service, flexible energy-storage devices are continuously subjected to complex external loads such as bending, stretching, compression and puncture. Quantitative deformation tests can evaluate both the intrinsic mechanical toughness of hydrogel matrices and the bonding reliability at electrode–gel interfaces. Common evaluation indicators include bending angle, tensile strain and cyclic loading times [95,96,97].

For bending performance, SA-based gel devices display divergent bending resistance. Qin et al. [98] fabricated SA/PAAm-hydrogel-based supercapacitors that retain 83.6% initial areal-specific-capacitance after 500 cycles of 180° bending. Yang et al. [99] constructed zwitterionic-hydrogel-based safe supercapacitors with above-90% capacitance retention after 100 bending cycles. Liu et al. [100] prepared catechol-alkali-metal-ion self-catalytic composite-gel devices maintaining 89.7% capacitance after 2000 repeated 180° bending–release cycles.

Tensile performance represents a key indicator for flexible devices. SA-based double-network hydrogels achieve fracture strain above 2000%, originating from multiple dynamic non-covalent bonds and PAAm interpenetrating networks [101,102]. Guo et al. [96] prepared hydrogel fibers with only 17.5% energy loss after 100 cycles of 1500% ultimate tensile–release cycles. Charge–discharge curves of partial devices almost overlap under 0–800% large tensile strain with negligible capacity decay [103]. Under cyclic-tensile tests, residual strains reach merely 1.2% and 4.5% at 100% and 200% strain respectively, with hysteresis coefficients higher than 98.2% [95]. Gel resistance fluctuates slightly and conductive networks remain intact after thousands of repeated tensile cycles [102]. CV and GCD test curves stay stable under low strain of 50% and 100% [104]. Long et al. [105] reported salt-soaking-modified PVA/SA conductive hydrogels with tensile strength up to 0.58 MPa.

For harsh deformation conditions such as compression and puncture, SA-based gels show certain tolerance. Electrochemical test curves have no obvious offset under multi-stage compression deformation of 50%, 65% and 75% [103]. After compression under a 200 g static load, areal specific capacitance rises to 126.92% of the initial value [106]; no obvious hysteresis appears over 20 compression–release cycles at a 50% compression ratio [99]; gel resistance remains stable during thousands of loading–unloading cycles [102]. In puncture-damage simulation tests, devices retain 79% capacitance under high-density puncture of 800 holes·cm^−2^ [103].

Deformation durability of gel devices results from the combined effect of intrinsic fracture energy of matrices and electrode–gel interfacial adhesion. SA composite hydrogels realize strong adhesion via multiple interactions including hydrogen bonds and metal coordination. Adhesion strength reaches 46.4 kPa on glass substrates and 2.16 MPa on wood substrates, effectively inhibiting electrode–gel delamination under deformation [94]. In contrast, internal debonding damage occurs inside gels when cohesive energy of materials is insufficient [100]. High-fracture-energy hydrogel matrices dissipate external impact through dynamic fracture-reconstruction of non-covalent bonds. Combined with robust interfacial adhesion, they jointly guarantee stable electrochemical output of flexible energy-storage devices under compound external-force deformation.

In real-world wearable scenarios, flexible energy-storage devices endure complex external loads including repeated bending, stretching, compression and puncture. Capacitance retention and deformation tolerance limits vary greatly for different SA-based hydrogel devices. Key mechanical–electrochemical test results are summarized in Table 7 for an intuitive horizontal comparison of comprehensive mechanical reliability of various devices.

As demonstrated in Table 7, two core prerequisites for maintaining electrochemical performance under deformation service conditions of flexible devices are high-fracture-energy gel matrices and robust electrode–gel interfacial adhesion. High intrinsic gel toughness alone cannot guarantee device stability. Even intact gel matrices will suffer sharp-rising contact impedance and rapid capacity fading once interfacial bonding fails. Although SA-based gels achieve satisfactory results in laboratory deformation tests, practical wearable applications still face urgent challenges such as long-term mechanical fatigue and adhesion degradation under humid-hot environments.

## 5. Existing Challenges and Future Development Directions: Dual-Track Pathways Toward Molecular Design and Industrialization

Although SA-based hydrogels have achieved multi-scale performance breakthroughs in flexible zinc-ion batteries, multi-level core contradictions remain from fundamental molecular design to large-scale industrial implementation. This chapter sorts out bottlenecks from molecular-theoretical design and industrial-manufacturing perspectives and puts forward a coordinated integrated development paradigm to provide clear development ideas for subsequent research.

### 5.1. Core Challenges at the Molecular-Design Level: Absence of Cross-Scale Structure–Performance Correlation and Shortage of Rational-Design Tools

At present, most modification studies for SA adopt trial-and-error experimental screening instead of quantitative and predictable rational-molecular-design systems, presenting two major shortcomings. First, cross-scale structure–property correlation models are lacking. Existing studies separate molecular functional groups, mesoporous structures and macro-device layers. Quantitative relationships cannot be established among carboxyl substitution degree, cross-linking density, filler-doping content and key indicators including ionic conductivity, dendrite-suppression capacity and wide-temperature stability. Theoretical parameters such as desolvation energy and interfacial-adsorption energy can hardly guide the regulation of gel formulas, which results in long development cycles and high screening costs for modified schemes [19]. Second, multi-scale simulation-aided design tools are imperfect. Current DFT and molecular-dynamics simulations are mostly confined to single-molecule coordination models and have not been extended to coupling simulations of three-dimensional gel networks and complete battery devices. High-throughput screening platforms based on machine learning are absent, which makes it impossible to predict comprehensive performances of combinations of different cross-linkers, salt systems and co-solvents in batches. Hence, modification strategies lack theoretical prediction support [15]. Meanwhile, existing modification approaches can hardly simultaneously satisfy multiple requirements including high-ion conduction, strong mechanical toughness, long-term water retention and extreme low-temperature tolerance. Inherent performance trade-offs make it difficult to optimize multiple indicators synchronously.

### 5.2. Key Barriers for Industrialization: Green-Synthesis Processes, Batch Consistency and Cost Control

Laboratory-scale small-batch preparation procedures cannot be directly scaled-up to industrial production lines. Three practical restrictions exist for large-scale manufacturing.

First, synthesis workflows are lengthy with high energy consumption and solvent loss. Traditional preparation includes multiple steps such as raw-material dissolution, long-time stirring, repeated centrifugal washing, freeze-drying and solvent replacement. Some systems even require high-temperature carbonization treatments. The whole production cycle is prolonged with high energy consumption, and substantial organic solvents raise environmental-post-treatment costs [42].

Second, there is a large batch-to-batch performance dispersion. The purity of industrial brown-algae raw materials fluctuates, leading to unstable carboxyl content. Cross-linkers and nano-fillers are difficult to disperse uniformly in large-volume solutions, resulting in obvious batch deviations in pore-structure and ionic conductivity of final gels. After device assembly, capacity and cycle life consistency are poor, which fails to meet requirements for standardized mass production [29].

Third, there are conflicts between cost and performance. High-end nano-fillers such as MXene and ZIF-8 greatly improve electrochemical performance yet come with high raw-material prices. Conventional cross-linking agents such as borax and heavy-metal salts are subject to environmental protection restrictions, and industrial-scale processes for green low-cost cross-linking systems remain immature [3]. Besides, existing coating and fiber-spinning forming equipment have poor compatibility. Large-area continuous fabrication of uniform ultra-thin gel electrolytes is difficult, restricting the mass-production scale of flexible fabrics and micro-devices.

Beyond the above-mentioned manufacturing-related barriers, a critical reassessment of the sustainability and conditional green credentials of SA-based hydrogel systems is needed. Even though the sodium-alginate matrix itself is a biodegradable marine-derived biomass polymer, the overall environmental friendliness of composite hydrogel electrolytes cannot be taken for granted. Apart from the already-discussed raw-material batch fluctuation, energy-intensive freeze-drying procedures, and high-cost advanced nanofillers, several additional practical concerns compromise its claimed green property. First, high-salt hydrogel formulations bring risks of waste-liquid discharge and salt residue upon disposal. Second, mature solvent recovery strategies are rarely reported for laboratory-level hydrogel synthesis, and organic antifreeze co-solvents are hard to recycle during gel forming. Third, end-of-life management remains largely underexplored. Pure SA hydrogel can biodegrade under natural conditions, yet composite hydrogels incorporated with inorganic nanofillers, cross-linking agents and abundant salts exhibit complex degradation profiles, and residual components may pose potential environmental hazards. Consequently, the “green” label should only be applied conditionally to SA-based hydrogel energy-storage systems and cannot be indiscriminately extended to the whole composite device.

### 5.3. Construction of “Structure-Function-Process” Integrated-Design Paradigm

Targeting dual bottlenecks of molecular design and industrialization, this paper proposes a three-in-one collaborative-development paradigm to bridge the technical chain from fundamental theory to engineering application.

Precise molecular-programming level: Build high-throughput screening platforms for functional groups, cross-linkers and fillers relying on DFT and machine-learning. Quantitatively regulate carboxyl exposure and cross-linking-site distribution of SA and directionally reconstruct Zn^2+^ solvation sheaths. Alleviate performance-trade-off contradictions from the molecular source to realize synchronous improvement of multiple indicators.

Closed-loop-process-regulation level: Develop room-temperature, one-step forming, continuous-flow coating/spinning new processes. Adopt green cross-linking agents such as polyphenols and biological ligands to replace borax and heavy-metal salts. Compound with agricultural-forestry straw and aquatic-processing by-products to cut raw-material costs, simplify high energy consumption procedures including washing and freeze-drying, and shorten production cycles.

Device-performance-mapping level: Establish quantitative mapping databases among gel mesoporous-cross-linking-structure parameters and battery energy density, cycle life and bending stability. Reverse-customize gel formulas and preparation processes according to device-application scenarios and realize demand-oriented forward material development.

This paradigm realizes two-way linkage among molecular simulation, pilot-scale processes and device testing. It solves bidirectional pain points: outstanding laboratory performance yet poor manufacturability, and industrial products failing performance standards. It promotes the large-scale commercialization of biomass-based energy-storage gels derived from SA.

## 6. Conclusions

Aqueous flexible energy-storage represents a key technical direction supporting next-generation wearable electronic equipment. Developing gel electrolytes that take into account high safety, excellent mechanical properties and electrochemical service performance lies at the core of breaking bottlenecks restricting its practical application. This review systematically summarizes the research progress of SA-based multifunctional hydrogel electrolytes for aqueous flexible energy-storage systems. It illustrates the unique structural advantages of SA derived from the G-block “egg-box” chelating cross-linking network. Such a structure can provide abundant tunable carboxyl active sites, realize the regulation of ion coordination, interface modification and zinc-deposition behavior at the molecular scale, and synergistically optimize the mechanical toughness and ion-transport capacity of hydrogels. Three major modification paradigms are concluded in this paper: multi-crosslinked network construction, functional-filler composite, and electrolyte-component regulation. The functional positioning and inherent performance trade-offs of different modification routes are distinguished. Multi-crosslinking enhances hydrogel mechanics and network stability; functional-filler composite builds electronic pathways and realizes interface functionalization; electrolyte-component engineering reconstructs Zn^2+^ solvation-sheaths to broaden the electrochemical window and improve wide-temperature-range adaptability. The synergistic combination of multiple strategies serves as an effective approach to reconcile various performance conflicts. By establishing a multi-scale coupling framework of molecular level–mesopore–electrode interface–device output, zinc-ion desolvation, optimized interfacial wettability and homogenized interfacial electric fields jointly suppress zinc-dendrite proliferation as well as corrosion-induced hydrogen-evolution side reactions, providing a unified analytical thinking for understanding the electrochemical behaviors of such hydrogels. Lab-scale device investigations demonstrate that SA-based hydrogels are compatible with diverse flexible configurations including carbon-based supercapacitors, zinc-ion hybrid energy storage, fibrous micro-energy-storage, and integrated energy-sensing devices, showing promising potential for future practical applications.

Nevertheless, this system still confronts numerous practical obstacles. Current modification strategies lack quantitative cross-scale structure–performance models and rational-design tools, and there exist intractable performance trade-offs among indicators such as mechanical properties, ionic conduction, water retention and anti-freezing capability. Meanwhile, prospective industrial transformation would be constrained by engineering challenges including raw-material quality fluctuation, insufficient processing compatibility and poor batch-to-batch device consistency. For future development, the integrated research paradigm of “precise molecular programming–closed-loop process regulation–reverse mapping of device performance” should be implemented. Multi-scale theoretical simulations and high-throughput screening shall be adopted to achieve targeted molecular modification. Green and facile preparation processes need to be developed to balance performance, cost and manufacturability. An evaluation system close to real-world service conditions should be constructed, and reliability verification of devices under complex environments should be strengthened, so as to fully explore the application value of eco-friendly biomass materials represented by SA in the field of next-generation safe and green flexible energy storage.

## Figures and Tables

**Table 1:** Comprehensive performance comparison of three mainstream biomass-hydrogel matrices.

Comparison Items	SA	Cellulose	Chitosan
Characteristic functional groups	High-density −COO^−^ carboxyl groups [19]	Abundant −OH hydroxyl groups (weak hydrogen-bond interaction only) [20]	−NH_2_ amino groups [21]
Cross-linking modes	Ion-chelated “egg-box” structure, dynamic supramolecular cross-linking [22]	Hydrogen bonds, chemical cross-linking; no ion-coordination sites [23]	Acid-base ionic cross-linking [21]
Zinc-dendrite-inhibition capacity	Carboxyl groups trap Zn^2+^ and homogenize interfacial electric fields [19]	No specific chelating sites; uneven ion distribution [23]	Amino groups tend to induce local charge accumulation [24]
Tensile toughness	Maximum tensile strain up to 2400% [18]	Brittle; fracture strain below 600% [25,26]	Moderate; prone to brittleness after drying [27]

**Table 2:** Cross-system performance comparison of representative aqueous flexible energy-storage configurations across lithium-ion, sodium-ion and zinc-ion chemistries, including typical hydrogel-electrolyte and liquid-electrolyte systems.

System Category	Materials (Electrode & Electrolyte)	Cycle Life	Core Distinctions from Hydrogel-Electrolyte Systems	Ref.
Na-ion(hydrogel-electrolyte-based)	Na metal anode, Na_3_V_2_(PO_4_)_3_ cathode; in-situ constructed vinyl–COF asymmetric flame-retardant composite gel electrolyte	full-cell 550 cycles (0.2 C, 99.6% capacity retention)	Covalently crosslinked COF nanochannels homogenize Na^+^ transport and suppress dendrites, featuring high modulus, flame resistance and stable asymmetric interfaces; organic plasticizer restricts wearable stretchability.	[31]
Li-ion(hydrogel-electrolyte-based)	Activated carbon electrodes; PPG-Li (PAM/peach-gum polysaccharide/glycerol/LiCl) multifunctional hydrogel electrolyte	20,000 cycles (25 °C, capacitance retention 94.2%)	Wide-temperature adaptability (−40–70 °C); intrinsic flame retardancy and self-adhesion; realizes integrated strain–sensor–supercapacitor system; limited energy density for lithium-based aqueous supercapacitor configuration	[32]
Zn-ion(hydrogel-electrolyte-based)	Zn anode; ZIF-8@PAM/SA composite hydrogel electrolyte	>1100 cycles	Dual-network structure for high stretchability; multifunctional sensing function; complicated nanofiller fabrication	[3]
Zn-ion(liquid-electrolyte-based)	Zn anode, Na_3_V_2_(PO_4_)_3_ cathode; anhydrous halozincate ionic liquid electrolyte	Zn full-cell 1000 cycles (80 °C, 76.9% capacity retention)	Anhydrous ionic liquid suppresses water-triggered hydrogen evolution and achieves dendrite-free Zn plating up to 120 °C with recyclable electrolyte; yet the rigid liquid cannot satisfy wearable mechanical demands.	[33]

**Table 3:** Comparison of electrolyte composition (salt species, concentration and co-solvent) on ionic conductivity, electrochemical window and temperature adaptability of SA-based hydrogels.

Main Components	Ionic Conductivity	Cell Configuration	Test Temperature	Current Density	Ref.
SA-g-DA/borax/3 M KCl	119.7 mS·cm^−1^ (25 °C)	AC‖hydrogel‖AC	−10 ~ 25 °C	1 A⋅g^−1^	[22]
MX-GO/CNFs/SA/PVA triple-network organohydrogel (MXene-graphene oxide conductive fillers)	Not reported	CNT film‖MGCSP organohydrogel‖CNT film (flexible all-solid-state SC)	25 °C	0.2 A⋅g^−1^	[60]
SAM–Zn (SA-MA with ZnSO_4_/ZnBr_2_ redox additive)	Not reported	Zn‖SAM–Zn‖AC	25 °C	2 A⋅g^−1^	[18]
PMAZn zwitterionic double-network hydrogel (PMS zwitterionic monomer + SA + ZnSO_4_)	32 mS·cm^−1^ (25 °C)	Zn‖PMAZn‖AC (zinc-ion hybrid supercapacitor); Zn‖PMAZn‖VS_2_ (zinc-ion battery)	25 °C	0.5 A⋅g^−1^	[66]
GG/SA/EG composite organohydrogel (guar gum + sodium alginate + ethylene glycol)	25.37 mS·cm^−1^ (25 °C)	Zn‖GG/SA/EG‖MnO_2_ (flexible solid-state zinc-ion battery)	−20 ~ 25 °C	0.15 A⋅g^−1^	[67]

**Table 4:** Comparison of optimization strategies for electrode-hydrogel interfacial compatibility.

System	Modification Strategy	Characterization Evidence	Interfacial Performance	Ref.
SAL-type SA hydrogel	Hydrogen bonding & metal coordination for full Zn surface wetting; uniform Cl^−^ distribution	FTIR/XPS peak shifts; DFT proves enhanced water binding energy	Confine free water; 92.3% capacity retention after 300 bending cycles	[12]
SA/CMC/THL ternary hydrogel	3D porous network; Zn-O coordination; occupy HER active sites on Zn	Uniform EDS element distribution; better wettability than liquid ZnSO_4_; FTIR/XPS Zn-O peaks	Zn^2+^ transference number = 0.70; suppressed concentration polarization	[53]
Gel/SA-acetate double network	Reconstruct Zn^2+^ solvation sheath via Zn^2+^–COO^−^ coordination	ATR-FTIR blue shift; MD/DFT: Zn^2+^ coordination number 6 → 3, low desolvation energy	Lower desolvation barrier, fast ion migration	[19]
PAM/SA composite hydrogel	Tridentate coordination between SA and Zn^2+^	FTIR peak shift; DFT confirms tridentate binding	Zn^2+^ transference number = 0.86, ionic conductivity = 5.4 S·m^−1^	[14]

**Table 5:** Comparison of zinc dendrite-suppression behaviours in SA-based hydrogel systems.

System	Suppression Mechanism	Test & Simulation Evidence	Suppression Effect	Ref.
SA with carboxyl anchoring	Carboxyl groups anchor Zn^2+^ to homogenize interfacial ion distribution	Deduced from ex-situ spectra and cycling data	Fundamental structural improvement for dendrite inhibition	[14]
SPS-Zn zwitterionic hydrogel	Preferential adsorption on Zn (002); inhibit lateral Zn^2+^ diffusion	DFT adsorption energy; CA test; in-situ optical observation; electric field simulation	Dendrite-free Zn deposition; 380 h symmetric cell cycling; 12,000 full-cell cycles	[15]
SA/CMC/THL crosslinked hydrogel	Crosslinked network locks free water; restrain corrosion & dendrite penetration	TGA reduced water loss at low temperature	3224 h stable Zn‖Zn cycling; 99.42% average CE for Zn‖Cu cell	[53]

**Table 6:** Comprehensive-performance comparison of flexible energy-storage devices constructed with SA-based hydrogels.

Main Components	Cell Configuration	Test Temperature	Current Density	Normalization Basis	Ref.
All-alginate-derived quasi-solid-state supercapacitor (NPC-2 electrode + -hydrogel electrolyte)	NPC-2‖-‖NPC-2 symmetric quasi-solid-state supercapacitor	25 °C	10 A⋅g^−1^	gravimetric	[72]
Gel/SA-acetate carboxylated double-network hydrogel electrolyte	Zn‖Gel/SA-acetate‖porous-carbon cathode zinc-ion hybrid supercapacitor	25 °C	20 A⋅g^−1^	gravimetric	[19]
SPMA-Zn powder self-healable SA-PMA composite hydrogel electrolyte	Zn‖SPMA-Zn‖AC zinc-ion hybrid supercapacitor (SPMA-ZHS)	25 °C	10 A⋅g^−1^	gravimetric	[82]
SAP dual cross-linked SA-PAAm hydrogel electrolyte (EG anti-freezing)	Zn@CC‖SAP-1.5‖AC@CC flexible zinc-ion hybrid capacitor	25 °C	1 A⋅g^−1^	gravimetric	[46]
Gel-Agar-SA-LiCl organohydrogel (gelatin-agar-sodium alginate with glycerol-water binary solvent, 3D-printable, highly adhesive)	AC‖Gel-Agar-SA-LiCl‖AC flexible symmetric supercapacitor integrated with multifunctional strain-temperature-humidity sensor	25 °C	1 A⋅g^−1^	gravimetric	[94]

**Table 7:** Summary of mechanical reliability tests for SA-based hydrogel flexible devices.

Test Type	Device/Gel System	Test Condition	Core Test Result	Ref.
Bending test	SA-PAAm flexible supercapacitor	500 cycles of 180° bending	83.6% retention of initial areal specific capacitance	[98]
Bending test	Catechol-modified SA adhesive-hydrogel device	2000 cycles of 180° bending-release	89.7% capacitance retention	[100]
Tensile test	Bionic octopus-tentacle porous-SA hydrogel device	100 cycles of 1500% ultimate tensile-release	17.5% energy loss, hysteresis ratio > 98.2%	[95]
Compression-loading test	PANI-PAAm/SA gel device	200 g static compressive load	Areal capacitance rises to 126.92% of initial value	[106]
Puncture test	High-toughness all-SA gel device	800 holes·cm^−2^ high-density puncture	79% capacitance retention	[103]
Puncture test	SA composite-gel supercapacitor	200 holes·cm^−2^ puncture	93.23% areal-capacitance retention	[106]
Self-healing & adhesion	Gelatin-SA strongly adhesive hydrogel device	Multiple fracture-rejoining self-healing	46.4 kPa adhesion strength (glass)	[94]

## Data Availability

No new data were created or analyzed in this study. Data sharing is not applicable to this article.

## References

[1] Kasprzak D., Mayorga-Martinez C.C., Pumera M. (2023). Sustainable and Flexible Energy Storage Devices: A Review. Energy Fuels.

[2] Wang Y., Huang Z., Li C., Dai O., Li M., Liu C., Hong W., Lei X., Wei H., Zhou T. (2025). Design and applications of antifreeze polysaccharide-based hydrogels for cryoprotection and biotechnological advancements: A review. Int. J. Biol. Macromol..

[3] Potu S., Gollapelli B., Gupta D. (2026). Metal–Organic Framework–Integrated Conductive Double-Network Hydrogels for Wearable and Self-Powered Electronic Systems. Adv. Sustain. Syst..

[4] Ye T., Li L., Zhang Y. (2020). Recent Progress in Solid Electrolytes for Energy Storage Devices. Adv. Funct. Mater..

[5] Rahman M.M., Hossen S., Rahman M.T., Sarkar B., Lee S.C., Jung Y., Shim J.S. (2025). High density 3D-structured graphene for long-life and high energy density lithium-ion battery. J. Power Sources.

[6] Rahman M.M., Rahman M.T., Hossen S., Sarkar B., Lee S.C., Jung Y., Shim J.S. (2025). Polyethersulfone-based thick polymer-supported graphene sheet for high energy density lithium-ion battery. J. Power Sources.

[7] Hossen S., Rahman M.M., Rahman M.T., Sarkar B., Devnath R., Shim J.S. (2026). Laser-induced graphene encapsulated-SiO_x_ hybrid anode with enhanced initial coulombic efficiency via prelithiation for long-life lithium-ion batteries. Chem. Eng. J..

[8] Hossen S., Rahman M.T., Rahman M.M., Sarkar B., Devnath R., Shim J.S. (2026). Single-step fabrication of a silicon suboxide–graphite-graphene hybrid anode with high reversible capacity and long cycling stability for lithium-ion batteries. J. Energy Storage.

[9] Ding Y., Xia H., Ding B., Wang S., Zhao X., Li G., Chen S., Hong X., Zhou X., Zhao Z. (2026). Flexible textile-based aqueous zinc-ion batteries: From energy storage mechanisms to wearable device integration. J. Alloys Compd..

[10] Weng G., Yang X., Wang Z., Xu Y., Liu R. (2023). Hydrogel Electrolyte Enabled High-Performance Flexible Aqueous Zinc Ion Energy Storage Systems toward Wearable Electronics. Small.

[11] Fu K., Zhang X., Lu X., He A., Chen L., Liu Y., Yang F., Zhou C. (2025). Self-healing hydrogels in flexible energy storage devices: Mechanisms, applications, and prospects. J. Mater. Chem. A.

[12] Yu Y., Huang G., Miao H., Liang J., Zhang X., Liu Y., Tong L., Fu X., Ge M., Qian Y. (2025). Confined Water Molecules of Ionic Conductive Hydrogel Electrolyte for the High-Performance, Dendrite-Free, and Safe Flexible Supercapacitor. ACS Appl. Energy Mater..

[13] Qi Z., Ren R., Hu J., Chen Y., Guo Y., Huang Y., Wei J., Zhang H., Pang Q., Zhang X. (2024). Flexible Supercapacitor with Wide Electrochemical Stable Window Based on Hydrogel Electrolyte. Small.

[14] Xiang Z., Li Y., Cheng X., Yang C., Wang K.-P., Zhang Q., Wang L. (2024). Lean-water hydrogel electrolyte with improved ion conductivity for dendrite-free zinc-Ion batteries. Chem. Eng. J..

[15] Wu T., Ji C., Zhang B., Mi H., Guo F., Guo G., Wu M. (2022). Construction of zwitterionic osmolyte-based hydrogel electrolytes towards stable zinc anode for durable aqueous zinc ion storage and integrated electronics. J. Mater. Chem. A.

[16] Zhang B., Cai X., Li J., Zhang H., Li D., Ge H., Liang S., Lu B., Zhao J., Zhou J. (2024). Biocompatible and stable quasi-solid-state zinc-ion batteries for real-time responsive wireless wearable electronics. Energy Environ. Sci..

[17] Quan Y., Ma H., Chen M., Zhou W., Tian Q., Han X., Chen J. (2023). Salting-Out Effect Realizing High-Strength and Dendrite-Inhibiting Cellulose Hydrogel Electrolyte for Durable Aqueous Zinc-Ion Batteries. ACS Appl. Mater. Interfaces.

[18] Han L., Miao H., Ni M., Zhao G., Zhu G., Li Y., Gao G., Huang H., Xu M. (2023). Functionally integrated self-healable and redox bromide-ion additive hydrogel electrolyte for Zn-ion hybrid supercapacitors. Mater. Lett..

[19] Tian C., Wang J., Sun R., Ali T., Wang H., Xie B.-B., Zhong Y., Hu Y. (2023). Improved Interfacial Ion Migration and Deposition through the Chain-Liquid Synergistic Effect by a Carboxylated Hydrogel Electrolyte for Stable Zinc Metal Anodes. Angew. Chem. Int. Ed..

[20] Liu Z., Wang R., Ma Q., Kang H., Zhang L., Zhou T., Zhang C. (2022). Application of cellulose-based hydrogel electrolytes in flexible batteries. Carbon Neutrality.

[21] Zhen S., Song Y., Xu J., Li X., Luo J., Xie Y., Ye J., Wu Y., Wang G., Qu Q. (2026). Anchorage-Capture Dual Mechanism in a Biomass-Derived Hydrogel Electrolyte for Dendrite-Free Aqueous Zinc Ion Batteries. Polymers.

[22] Tao F., Qin L., Wang Z., Pan Q. (2017). Self-Healable and Cold-Resistant Supercapacitor Based on a Multifunctional Hydrogel Electrolyte. ACS Appl. Mater. Interfaces.

[23] Chen F., Li X., Chen S.-P., Zhang Y., Huang H.-D., Yang H., Zhou S., Li Z.-M. (2025). Tough Cellulose Hydrogel Electrolyte with Low Solvation for Highly Reversible and Flexible Aqueous Zinc-Ion Battery. Adv. Sci..

[24] Kong Y., Li J., Xie B.-B., Chen J., Shen H., Wang H., Hu Y. (2026). Polysaccharide-based hydrogels and additives in electrolyte engineering for Zn-ion batteries: Anode stabilization mechanisms, performance enhancement, and perspectives. Chem. Commun..

[25] Zeng H., Wang C., Liu M., Li H., Ma J. (2026). Ion-solvation-mediated structure-preserving regeneration of colored waste cotton fabrics for the construction of cellulose-based gel electrolytes. Chem. Eng. J..

[26] Huang X., Ao X., Yang L., Ye J., Wang C. (2023). Preparation and properties of cellulose nanocrystal-based ion-conductive hydrogels. RSC Adv..

[27] Zheng Q., Shang M., Li X., Jiang L., Chen L., Long J., Jiao A., Ji H., Jin Z., Qiu C. (2024). Advances in intelligent response and nano-enhanced polysaccharide-based hydrogels: Material properties, response types, action mechanisms, applications. Food Hydrocoll..

[28] Adhyapak H.M., Prakash A., Holla S.R., De S. (2026). Unleashing supercapacitor potential via carbon nano onions infused with nickel oxide microspheres and hydrogel electrolyte. J. Mater. Sci. Mater. Electron..

[29] Zhang R., Wu C., Yang W., Yao C., Jing Y., Yu N., Su S., Mahmud S., Zhang X., Zhu J. (2024). Design of delignified wood-based high-performance composite hydrogel electrolyte with double crosslinking of sodium alginate and PAM for flexible supercapacitors. Ind. Crops Prod..

[30] Tong H., Xing Z., Wang Y., Li Z., Li S., Zhang Q. (2026). Imidazolium ionic liquid-endowed polyacrylamide/sodium alginate carbon-based hydrogels with a high voltage window and high sensitivity for wearable flexible electronic devices. Chem. Eng. J..

[31] Chen S., Miao Z., Guo J., Che H., Ma Z.-F., Liu T. (2026). Covalently crosslinked nanoconfinement strategy driven asymmetric flame-retardant gel electrolytes toward dendrite-free sodium metal batteries. J. Energy Chem..

[32] Jiang M., Ye L., Huang Y., Wang W., Zhang Z., Lu T., Lu S. (2026). Integrated system of a strain sensor and flexible supercapacitor based on a multifunctional hydrogel electrolyte. Chem. Eng. J..

[33] Yang M., Zou X., Wu M., Yu J., Ma X., Hu Y., Yan F. (2025). Halozincate ionic liquid electrolyte enabled high-temperature dendrite-free Zn metal batteries. Energy Environ. Sci..

[34] Huang B., Liu W., Lan Y., Huang Y., Fu L., Lin B., Xu C. (2024). Highly ion-conducting, robust and environmentally stable poly(vinyl alcohol) eutectic gels designed by natural polyelectrolytes for flexible wearable sensors and supercapacitors. Chem. Eng. J..

[35] Pei J., Chen G., Li Z., Zhou Z., Chen A., Xie S., Jiang X. (2023). Electricity Generation with Sodium Alginate Hydrogel for Osmotic Energy Harvesting. Ind. Eng. Chem. Res..

[36] Lei J., Zeng X., Tang X., Wen J., Liu J., Guo S., Dai X., Wu F., Xue A. (2026). Zinc Citrate Chelate Derivative-Modified Sodium Alginate Hydrogel Electrolyte with Controlled Water Activity for Ultrastable Zn Anodes. ACS Appl. Mater. Interfaces.

[37] Wang P., Han P., Wan R., Gao P., Geng W., Meng Z., Zhang C. (2025). Compatibility electrode-electrolyte interface through supramolecular gel polymer electrolyte for flexible energy storage. Polym. Test..

[38] Zheng J., Yu X., Wang C., Cao Z., Yang H., Ma D., Xu X. (2016). Facile synthesis of three-dimensional reinforced Sn@polyaniline/sodium alginate nanofiber hydrogel network for high performance lithium-ion battery. J. Mater. Sci. Mater. Electron..

[39] Sun H., Zhou X., Tan M., Yang D., Wan W., Li Y., Yao R., Zhang J., Liu S., Lang X. (2025). Tin disulfide coordinated with sodium alginate as stable anode materials for high-performance lithium ion batteries. J. Energy Storage.

[40] Saadi-motaallegh S., Omidvar H. (2026). Polyethylene Glycol Diacid–Engineered Semi-Interpenetrating Hydrogel Electrolyte for Durable Aqueous Zinc-Ion Batteries. ACS Appl. Energy Mater..

[41] Peng K., Wang W., Zhang J., Ma Y., Lin L., Gan Q., Chen Y., Feng C. (2022). Preparation of chitosan/sodium alginate conductive hydrogels with high salt contents and their application in flexible supercapacitors. Carbohydr. Polym..

[42] Shi H., Xie Y., Wei P., Chen H., Qiang Y. (2020). Application of Co-Mo bimetal/carbon composite in dye-sensitized solar cells and its research on synergy mechanism. J. Solid State Electrochem..

[43] Jiang P., Liu Q., Hao M., Duan N., Min Z., Yang J., Xu N., Ma S., Qin C. (2026). Sodium alginate-incorporated double-network polymer hydrogel electrolyte with synergistic self-healing and low-temperature resistant properties for energy storage devices. Chem. Eng. J..

[44] Li Y., Liu X., Gong Q., Xia Z., Yang Y., Chen C., Qian C. (2021). Facile preparation of stretchable and self-healable conductive hydrogels based on sodium alginate/polypyrrole nanofibers for use in flexible supercapacitor and strain sensors. Int. J. Biol. Macromol..

[45] Yu M., Gao Y., Hong H., Wang T., Peng Z. (2025). A versatile, highly stretchable, and anti-freezing alginate/polyacrylamide/polyaniline multi-network hydrogel for flexible strain sensors and supercapacitors. Int. J. Biol. Macromol..

[46] Tang Q., Kuang S., Yang S., Tong M., Zeng S., Yu X. (2025). Mechanically robust, anti-freezing, and dual cross-linked alginate-polyacrylamide hydrogel electrolyte for flexible zinc ion hybrid capacitors (ZHCs) with enhanced wide temperature range. Cellulose.

[47] Li T., Wei H., Zhang Y., Wan T., Cui D., Zhao S., Zhang T., Ji Y., Algadi H., Guo Z. (2023). Sodium alginate reinforced polyacrylamide/xanthan gum double network ionic hydrogels for stress sensing and self-powered wearable device applications. Carbohydr. Polym..

[48] Meng L., Liu D., Liu E., Wu Z., Xu M. (2025). Ion Cross-Linked High-Strength and Toughness Multinetwork Ionic Organic Hydrogels for Flexible Electronic Devices. ACS Appl. Electron. Mater..

[49] Yang T., Xu J., Lv S. (2024). HNTs Improve Flame Retardant and Thermal Insulation of the PVA/CA Composite Aerogel. ACS Omega.

[50] Hsiao Y., Lee L., Lin Y., Hong S., Wang K., Tung S., Liu C. (2023). Stretchable polyvinyl alcohol and sodium alginate double network ionic hydrogels for low-grade heat harvesting with ultrahigh thermopower. Mater. Today Energy.

[51] Li W., Yang S., Chen W., Yang J., Yu H., Lv R., Fu M. (2024). Free-standing and flexible polyvinyl alcohol-sodium alginate-polypyrrole electrodes based on interpenetrating network hydrogels. J. Colloid Interface Sci..

[52] Najafloo M., Naji L. (2025). The electrochemical and physicochemical impacts of sodium chloride on the formation of a self-healing double network hydrogel using two methods—Post-treatment and in situ integration. Sustain. Energy Fuels.

[53] Li X., Jiang Y., Li Y., Wang D., Ran F. (2025). Highly reversible zinc anode enabled by trehalose modified sodium alginate/carboxymethyl cellulose hydrogel electrolyte. Chem. Eng. J..

[54] Liu R., Zhao X., Liang L., Li R., Fan P., Li J., Zhao H. (2023). Double-network aerogel-derived spongy 3D porous MnO/C composite for lithium-ion batteries with increasing capacity. J. Alloys Compd..

[55] Chen W., Zhu S., Gu Y., Xiong F., Lu H., Wu X., Qin Z., Deng S., Yang B.-R. (2024). Polyvinyl alcohol/sodium alginate hybrid materials with high conductivity and transparency performance for woven electrode fibers. Compos. Sci. Technol..

[56] Wang Y., Yang H., Wang J., Dong J., Duan Y. (2023). Self-Supporting Conductive Polyaniline–Sodium Alginate–Graphene Oxide/Carbon Brush Hydrogel as Anode Material for Enhanced Energy in Microbial Fuel Cells. Coatings.

[57] Yin J., Liu Q., Zhou J., Zhang L., Zhang Q., Rao R., Liu S., Jiao T. (2020). Self-assembled functional components-doped conductive polypyrrole composite hydrogels with enhanced electrochemical performances. RSC Adv..

[58] Raza S., Bashir T., Chen C., Yu W., Ahmad M.T., Wu X., Hameed M.U., Shen L., Lin H., Orooji Y. (2025). Self-assembled in-situ growth of polyaniline on MXene composite with sodium alginate hydrogel 3D framework for enhanced LIBs performance. Int. J. Biol. Macromol..

[59] Shi G., Zhan T., Hu Y., Guo Z., Wang S. (2023). A stretchable, self-adhesive, conductive double-network hydrogel and its application in flexible strain sensors. J. Polym. Res..

[60] Hu K., Zhao Z., Wang Y., Yu L., Liu K., Wu H., Huang L., Chen L., Ni Y. (2022). A tough organohydrogel-based multiresponsive sensor for a triboelectric nanogenerator and supercapacitor toward wearable intelligent devices. J. Mater. Chem. A.

[61] Ke X., Yang F., Huang H., Zhao R., Huang J., Ji J., Li L. (2022). Stable polyaniline/sodium alginate/graphene oxide composite hydrogels as binder-free supercapacitor electrodes. Ionics.

[62] Li L., Cao M., Zhang D., Jia S., Yue S., Wang C., Qiu H., Ji Y. (2024). Hydrogel-stabilized zinc ion batteries: Progress and outlook. Green Chem..

[63] Li Z., Liang Z., Ma Z., Qu P., Zhang Y. (2024). Boosting the anode performance of the porous Si coated with polydopamine and cross-linked with sodium alginate. J. Alloys Compd..

[64] Li Z., Zhao D., Fu H., Wang A., Cao H., Zong P. (2024). Conductive copper ion-reinforced sodium alginate aerogel based free-standing Si anode for Li-ion storage. Solid State Ion..

[65] Zhao D., Wang A., Wang Z., Wang Y., Chen S., Zeng S., Zhang M., Zong P. (2025). Sodium alginate/chitosan double-network based high-performance free-standing silicon anodes. Electrochim. Acta.

[66] Chen Q., Zhao J., Chen Z., Jin Y., Chen J. (2022). High voltage and self-healing zwitterionic double-network hydrogels as electrolyte for zinc-ion hybrid supercapacitor/battery. Int. J. Hydrogen Energy.

[67] Wang J., Huang Y., Liu B., Li Z., Zhang J., Yang G., Hiralal P., Jin S., Zhou H. (2021). Flexible and anti-freezing zinc-ion batteries using a guar-gum/sodium-alginate/ethylene-glycol hydrogel electrolyte. Energy Storage Mater..

[68] Xu B., Ma Q., Cui Z., Li J., Li Z., Huo B., Li S., Sun D. (2024). Self-assembly of MoS_2_ nanoflakes contributing to continuous porous hydrogel for high-rate flexible zinc battery. J. Energy Storage.

[69] Mohd Said M.Z., Kamarulazam F., Farhana N.K., Manogran P., Bashir S., Ramesh S., Ramesh K. (2025). Influence of natural rubber content on the electrochemical performance of sodium alginate/acrylamide/natural rubber hydrogel electrolyte supercapacitors. J. Mater. Sci..

[70] Huang H., Zeng X., Li W., Wang H., Wang Q., Yang Y. (2014). Reinforced conducting hydrogels prepared from the in situ polymerization of aniline in an aqueous solution of sodium alginate. J. Mater. Chem. A.

[71] Fadavi P., Mehrpooya M., Ganjali M.R. (2026). Dual metal deposited MXene-Covalent organic framework hydrogel for supercapacitor and water splitting applications. J. Power Sources.

[72] Li Y., Yang L., Xiong R., Shang Y., Tian Y., Chi B., Han B., Xia K., Wang H., Wang Q. (2025). High Energy Quasi-Solid-State Supercapacitors Totally Derived from Alginate Hydrogel. Small.

[73] Badawi N., Aldayle M., Khalifa A. (2026). Ajwa Date Seed-Derived Hydrogel Electrolyte with Enhanced Electrochemical Performance and Mechanical Strength for Flexible Supercapacitors. Gels.

[74] Wang H., Liu J., Ahmed S., Wang T., Song S. (2022). Freeze-tolerant gel electrolyte membrane for flexible Zn-ion hybrid supercapacitor. J. Energy Storage.

[75] Darwish A., El-Sayed N.S., Al Kiey S.A., Kamel S., Turky G. (2023). Polyanionic electrically conductive superabsorbent hydrogel based on sodium alginate-g-poly (AM-co-ECA-co-AMPS): Broadband dielectric spectroscopy investigations. Int. J. Biol. Macromol..

[76] Wan H., Zhao X., Guo Q., Gao C., Sun R. (2025). Stretchable and conductive lignin hydrogel electrolyte for flexible supercapacitor. Front. Chem. Sci. Eng..

[77] Huang J., Zhang W., Huang H., Liu Y., Yang Q., Li L. (2019). Facile Synthesis of N,S-Codoped Hierarchically Porous Carbon with High Volumetric Pseudocapacitance. ACS Sustain. Chem. Eng..

[78] AL-Ghaus Z., Akbarinejad A., Zhu B., Travas-Sejdic J. (2021). Polyluminol-polyoxometalate hybrid hydrogels as flexible and soft supercapacitor electrodes. J. Mater. Chem. A.

[79] Ruthes J.G.A., Deller A.E., Pameté E., Riegel-Vidotti I.C., Presser V., Vidotti M. (2023). Hydrogel-Based Flexible Energy Storage Using Electrodes Based on Polypyrrole and Carbon Threads. Adv. Mater. Interfaces.

[80] Badawi M.N., Bhatia M., Ramesh S., Ramesh K., Khan M., Adil S.F. (2023). Enhancement of the Performance Properties of Pure Cotton Fabric by Incorporating Conducting Polymer (PEDOT:PSS) for Flexible and Foldable Electrochemical Applications. J. Electron. Mater..

[81] Feng T., Cao X., Li T., Diao R., Jing Y., Zhang X., Zhu J. (2025). Zinc-Ion–Cross-Linked Sodium Alginate-Wood Hydrogel Electrolyte for Supercapacitors via a Top-Down Design. Energy Technol..

[82] Liu T., Ren R., Qi Z., Hu J., Chen Y., Huang Y., Guo Y., Cao H., Liang M., Sun J. (2024). High-performance micro supercapacitor assembled by laser-induced graphene electrode and hydrogel electrolyte with excellent interfacial wettability for high capacitance. J. Power Sources.

[83] Huang H., Han L., Fu X., Wang Y., Yang Z., Pan L., Xu M. (2021). A Powder Self-Healable Hydrogel Electrolyte for Flexible Hybrid Supercapacitors with High Energy Density and Sustainability. Small.

[84] Liu B., Ma Y., Wang J., Kang X., He L., Lei L., Ran F. (2022). Fabrication of nickel cobalt bimetallic sulfide doped graphite carbon nanohybrids as electrode materials for supercapacitors. Diam. Relat. Mater..

[85] Badawi N.M., Batoo K.M., Subramaniam R., Kasi R., Hussain S., Imran A., Muthuramamoorthy M. (2023). Highly Conductive and Reusable Cellulose Hydrogels for Supercapacitor Applications. Micromachines.

[86] Hu Y., Liu C., Zhang H., Bao W., Wang F., Maimaitiyiming X. (2026). Agar-modified gelatin/polyvinyl alcohol-based tough hydrogels for 3D printing to prepare multifunctional sensors and flexible supercapacitors. Carbohydr. Polym..

[87] Zhou J., Zhang H., Zhang Q., Luo Y., Zang J., Wang Z., Li J., Ruzimuradov O., Mamatkulov S., Butanov K. (2026). All-weather high-performance fibrous supercapacitor based on graphene oxide/amino-functionalized metal-organic framework UiO-66/sodium alginate composite hydrogel electrolyte for wearable electronics. J. Energy Storage.

[88] Ling G., Ren J., Wang Z., Ren R.-P., Lv Y. (2024). Integrating self-hygroscopic hydrogel electrolyte and porous electrode for supercapacitor operating in variable humidity environments. J. Energy Storage.

[89] Peng K., Zhang J., Yang J., Lin L., Gan Q., Yang Z., Chen Y., Feng C. (2022). Green Conductive Hydrogel Electrolyte with Self-Healing Ability and Temperature Adaptability for Flexible Supercapacitors. ACS Appl. Mater. Interfaces.

[90] Najafloo M., Naji L. (2024). Resilient 3D porous self-healable triple network hydrogels reinforced with graphene oxide for high-performance flexible supercapacitors. J. Alloys Compd..

[91] Tian J., Sun Z., Shi C., Huang Z. (2023). Rapid fabrication of tough sodium alginate/MXene/poly(vinyl alcohol) dual-network hydrogel electrolytes for flexible all-solid-state supercapacitors. Int. J. Biol. Macromol..

[92] Ma Z., Zhao J., Fan Y., Qin X., Shao G. (2022). High surface area of crystalline/amorphous ultrathin MnO_2_ nanosheets electrode for high-performance flexible micro-supercapacitors. J. Alloys Compd..

[93] Hu O., Lu J., Chen G., Chen K., Gu J., Weng S., Hou L., Zhang X., Jiang X. (2021). An Antifreezing, Tough, Rehydratable, and Thermoplastic Poly(vinyl alcohol)/Sodium Alginate/Poly(ethylene glycol) Organohydrogel Electrolyte for Flexible Supercapacitors. ACS Sustain. Chem. Eng..

[94] Hu Y., Maimaitiyiming X. (2024). Gelatin/sodium alginate-based strongly adhesive, environmentally resistant, highly stable hydrogel for 3D printing to prepare multifunctional sensors and flexible supercapacitors. Int. J. Biol. Macromol..

[95] Shang Y., Liu C., Tian J., Zhou R., Hu Q., Sun X. (2025). Flexible and low-temperature-resistant double-network hydrogel with a bionic octopus-tentacle-like structure for integrated supercapacitor and nanogenerator sensor fabrication. J. Colloid Interface Sci..

[96] Guo J., Bao D., Zhang S., Zhang X., Ji X., Li M., Guan F. (2024). Sodium alginate-based high conductive, ultra-stretchable hydrogel fibers for electrolytes of flexible solid-state supercapacitors. Int. J. Biol. Macromol..

[97] Li B., Li J., Bi X., Tie A., Li J., Sun M., Li Z., Xu B. (2026). GO-induced in-situ polymerization of aniline on hydrogel electrolyte into one-body flexible supercapacitor for motion-sensing application. Electrochim. Acta.

[98] Qin C., Gao X., Wang D., Xiong H., Wu B. (2025). Flexible, Robust Sodium Alginate/PAAm Hydrogel for High-Performance Supercapacitor. J. Appl. Polym. Sci..

[99] Yang Z., Han L., Fu X., Wang Y., Huang H., Xu M. (2022). Double-safety flexible supercapacitor basing on zwitterionic hydrogel: Over-heat alarm and flame-retardant electrolyte. Adv. Compos. Hybrid Mater..

[100] Liu H., Liu J., Gao H., Ma F., Xin S., Ji X., Gai L., Jia G., Liu L. (2025). Catechol-alkali metal ion autocatalytic hydrogel electrolyte with strong adhesion and anti-freezing performances for flexible electronic devices. Chem. Eng. J..

[101] Liu X., Li W., Wang P., Xing Z. (2024). Stretchable and conductive polyacrylamide/sodium alginate dual-network hydrogels mediated by HNO_3_ for flexible capacitors and wearable sensors. New J. Chem..

[102] Liu J., Zhao W., Li J., Li C., Xu S., Sun Y., Ma Z., Zhao H., Ren L. (2024). Multimodal and flexible hydrogel-based sensors for respiratory monitoring and posture recognition. Biosens. Bioelectron..

[103] Zeng J., Dong L., Sha W., Wei L., Guo X. (2020). Highly stretchable, compressible and arbitrarily deformable all-hydrogel soft supercapacitors. Chem. Eng. J..

[104] Chen X., Shi C., Wang T., Wang G., Zhao Y., Liu Y., Zhang D. (2022). Polyacrylamide-Graphene Oxide-Polyaniline Based Flexible Electrode for Portable Supercapacitor. Macromol. Chem. Phys..

[105] Song C., Yu L., Liu H., Yang Z., Wu Y., Liu F., Zhang S., Gao S., Li M. (2024). Surfactant-assisted preparation of all-gel-state flexible supercapacitor with remarkable electrochemical performance based on polyaniline-polyacrylamide/sodium alginate hydrogels. Int. J. Biol. Macromol..

[106] Long K., Zhang Y., Gao X., Li J., Luo Y., Huang M., Mao Y., Hu C., Guo S. (2024). A Flexible, Conductive Hydrogel for Strain Sensor and Triboelectric Nanogenerator toward Human Motion Monitoring. ACS Appl. Electron. Mater..

